# Comparative Genomics Provide Insights Into Function and Evolution of Odorant Binding Proteins in *Cydia pomonella*

**DOI:** 10.3389/fphys.2021.690185

**Published:** 2021-07-07

**Authors:** Cong Huang, Xue Zhang, Dongfeng He, Qiang Wu, Rui Tang, Longsheng Xing, Wanxue Liu, Wenkai Wang, Bo Liu, Yu Xi, Nianwan Yang, Fanghao Wan, Wanqiang Qian

**Affiliations:** ^1^Shenzhen Branch, Guangdong Laboratory for Lingnan Modern Agriculture, Genome Analysis Laboratory of the Ministry of Agriculture and Rural Affairs, Agricultural Genomics Institute at Shenzhen, Chinese Academy of Agricultural Sciences, Shenzhen, China; ^2^College of Plant Health and Medicine, Qingdao Agricultural University, Qingdao, China; ^3^Hubei Engineering Research Center for Pest Forewarning and Management, Yangtze University, Jingzhou, China; ^4^Guangdong Key Laboratory of Animal Conservation and Resource Utilization, Guangdong Public Laboratory of Wild Animal Conservation and Utilization, Institute of Zoology, Guangdong Academy of Sciences, Guangzhou, China; ^5^Institute of Plant Protection, Chinese Academy of Agricultural Sciences, Beijing, China

**Keywords:** odorant binding proteins, codling moth, *Cydia pomonella*, positive selection, comparative genomics, gene gains and losses

## Abstract

Insect olfaction is vital for foraging, mating, host-seeking, and avoidance of predators/pathogens. In insects, odorant binding proteins (OBPs) are involved in transporting hydrophobic odor molecules from the external environment to receptor neurons. The codling moth, *Cydia pomonella*, one of the most destructive insect fruit pests, causes enormous economic losses. However, little is known about the number, variety, gains and losses, and evolution of *OBP* genes in *C. pomonella*. Here we report the identification of 40 *OBPs* in *C. pomonella*, most (75%) of which are classic *OBPs*, using genomic and transcriptomic analyses. Two *OBP* genes were lost in *C. pomonella* relative to possible distant ancestor in Lepidoptera lineage based on an analysis of gene gains and losses. The phylogenetic tree and chromosome location showed that the expansion of *OBP* genes mainly resulted from tandem duplications, as the *CpomGOBP2* gene was duplicated twice along with loss of *CpomPBPB*. Two positive selection sites of the *CpomGOBP1* gene were identified while other *OBP* genes evolved under purifying selection. Our results provide fundamental knowledge of *OBP* genes allowing further study of their function in *C. pomonella*.

## Introduction

Insects rely on their olfactory system to sense environmental odors related to behaviors such as foraging, host-seeking, mating, and oviposition, as well as avoiding predators and pathogens (Andersson et al., 2015). Odorant binding proteins (OBPs) are small water-soluble globular proteins with molecular masses of 10–30 kDa (Sun et al., 2018). *OBPs* are highly expressed in the hydrophilic lymph of insect olfactory sensilla (Pelosi et al., 2014). When lipophilic semiochemicals from the environment enter the lymph through micropores on the surface of olfactory sensilla, the *OBPs* bind, solubilize, and deliver the semiochemicals to the receptor proteins, e.g., odorant receptors (ORs) or ionotropic receptors (IRs), which are located on the membranes of olfactory sensory neurons. This delivery activates a series of downstream olfactory signal transductions accompanied by corresponding behavioral movements in insects (Zhang et al., 2020). *OBPs* are clearly essential in communications between insects and environmental semiochemicals including both pheromones and host volatiles.

*OBPs* are involved in the initial step of recognizing host volatiles or sex pheromones, suggesting that the functional divergence of *OBPs* is associated with speciation or host diversity. Considering the low sequence identities between orthologous/paralogous *OBP* genes, *OBP* genes have likely been evolving at a rapid rate through gene gains or losses (McKenzie et al., 2014) and positive selection (Campanini and de Brito, 2016). Most *OBP* genes are tandemly arranged in chromosomes, indicating that the occurrence of these genes arose from tandem duplication (Hekmat-Scafe et al., 2002; Gong D. P. et al., 2009; Manoharan et al., 2013; Dippel et al., 2014). The duplicate genes then gradually diverge in function through mutation or pseudogenization (Nei and Rooney, 2005; Vieira et al., 2007).

Studies on the origin, evolution, and structural variation of *OBP* genes provide insight into the functional differentiation of *OBPs* and host preference in insects. However, there is little knowledge of the numbers, structures, and evolution of the *OBP* gene family in important insect crop pests such as the codling moth, *Cydia pomonella* (L.) (Lepidoptera: Tortricidae). *C. pomonella* is an economically threatening pest worldwide (Witzgall et al., 2008; Kumar et al., 2015; Zhu et al., 2017). It mainly destroys apples and pears as well as other seed and stone fruits. Some studies focused on the structures and functions of pheromone binding proteins (PBPs) (Liu et al., 2016; Tian et al., 2016a, b; Tian and Zhang, 2016) and identification of general odorant binding proteins (GOBPs) (Garczynski et al., 2013). In contrast, studies on *OBPs* are lacking in *C. pomonella*, with little information of their roles in recognizing hosts or locating mates.

To understand the evolution and function of *OBPs* in *C. pomonella*, we identified and annotated its *OBP* genes by combining transcriptome data with the high quality genome we released previously (Wan et al., 2019). The gene gains and losses of *OBPs* were estimated by CAFÉ 3.0 (Han et al., 2013) for seven moth species. Subsequently, a phylogenetic tree of *OBP* genes from three lepidopteran insects (*C. pomonella*, *Bombyx mori*, and *Manduca sexta*) was constructed to explore their evolutionary relationships. The collinearity and chromosome locations were used to compare the divergence of *OBP* genes between *C. pomonella* and *B. mori*. Finally, the positive selection of genes and structural homology model were analyzed to predict the functional divergence of selected *OBPs*. Our results provide insights into the evolution of *OBP* genes in *C. pomonella*, which will facilitate future functional studies.

## Materials and Methods

### Identification of *OBP* Genes in the *C. pomonella* Genome

The protein sequences of seven lepidopteran insect *OBPs* were collected from deposited data of published articles, which have been identified from their genomes, and these species included *B. mori* (Gong D. P. et al., 2009), *M. sexta* (Vogt et al., 2015), *Plutella xylostella* (Cai et al., 2020), *Spodoptera litura* (Cheng et al., 2017), *Spodoptera frugiperda* (Gouin et al., 2017), *Helicoverpa armigera* (Pearce et al., 2017), and *Danaus plexippus* (Zhan et al., 2011). These protein sequences were then used as queries in iterative BLASTP searches with parameter “-*e-*value 1e–5” against the *C. pomonella* genome (Wan et al., 2019) to find candidate *OBP* genes. A local command line HMMER (version 3.1b2) search was conducted for these candidate *OBP* genes against the Pfam-A database (El-Gebali et al., 2019) to find the PBP_GOBP (PF01395) HMM profile. The identified *OBP* genes were subsequently used as queries to align the *C. pomonella* genome using tBLASTn search with parameter “*-e*-value 1e-5” to identify the missing *OBP* genes during gene prediction for the genome. We used an in-house Perl script to extract DNA sequences of novel genes from the genome, followed by predicting the CDS using the online website FGENESH (Solovyev et al., 2006). Gene prediction was verified by comparing with the transcriptome data that we used in the *C. pomonella* genome paper to confirm the complete gene structure (Wan et al., 2019). Finally, we used GMAP (Wu and Watanabe, 2005) to rebuild gene structures of all *OBP* genes. For *B. mori*, we used the 44 *OBPs* of *B. mori* which were identified by Gong (Gong D. P. et al., 2009), to perform tBLASTn search against the newest version of the *B. mori* genome (Kawamoto et al., 2019) and rebuilt their gene structures by GMAP (Wu and Watanabe, 2005).

To check the conserved cysteine pattern, which is the predominant feature of *OBP* genes, we first performed multiple sequence alignment of *OBP* sequences using MAFFT v7 (Katoh et al., 2002) with default parameters. Then, the aligned sequences were trimmed by trimAl v1.2 (Capella-Gutierrez et al., 2009) to remove gaps and low-quality regions with the parameter ‘‘-automated1.’’ The trimmed sequences were subsequently submitted to ESPript 3.0 Server^1^ for visualization.

### Estimation of Gene Gains and Losses

To explore gene gains and losses of *OBPs* in moths, seven moth species with available genomes and past investigations of the *OBP* gene family were selected, including *S. litura*, *S. frugiperda*, *H. armigera*, *B. mori*, *M. sexta*, *C. pomonella*, and *P. xylostella*. Orthologous and paralogous groups of these species were inferred by OrthoFinder v2.3.1 (Emms and Kelly, 2015) with default parameters. Orthologous groups including only single copy genes for each species were selected to construct the species tree. Protein sequences of each orthologous group were independently aligned using MAFFT v7 (Katoh et al., 2002), trimmed by trimAl v1.2 (Capella-Gutierrez et al., 2009), and then concatenated into one super-sequence. The phylogenetic tree was inferred using maximum likelihood (ML) in RAxML with the best-fit model (JTT + I + F) estimated by ProtTest3 v3.4.2 (Darriba et al., 2011). The Bayesian Relaxed Molecular Clock (BRMC) approach was adopted to estimate the neutral evolutionary rate and species divergence time using the program MCMCTree, implemented in PAML v4.9b package (Yang, 2007). The tree was calibrated with the following time frames adopted from TimeTree (Kumar et al., 2017) to constrain the age of the nodes between the species: 99–121 million years ago (Mya) for *B. mori* and *H. armigera*, and 80–243 Mya for *C. pomonella* and *P. xylostella*.

The *OBP* gene gains and losses were estimated by CAFÉ v3.0 (Han et al., 2013). Gene numbers of the *OBP* gene family in each insect were collected from published articles for *S. litura* (Cheng et al., 2017), *S. frugiperda* (Gouin et al., 2017), *H. armigera* (Pearce et al., 2017), *M. sexta* (Vogt et al., 2015), and *P. xylostella* (Cai et al., 2020), while the numbers of *OBP* genes in *B. mori* and *C. pomonella* were identified in this study (see section “Materials and Methods”). This gene number matrix together with the phylogenetic tree corrected by MCMCTree were used as input files for CAFÉ 3.0 (Han et al., 2013).

### Phylogenetic Analysis

A total of 133 *OBP* genes from three species (*C. pomonella*, *B. mori*, and *M. sexta*) were used in the phylogenetic analysis. These gene sequences were aligned using MAFFT v7 (Katoh et al., 2002) with default parameters, then the alignments were trimmed by trimAl v1.2 (Capella-Gutierrez et al., 2009) with the parameter “-automated1.” RAxML (Stamatakis, 2014) was used to construct a maximum likelihood evolutionary tree with the best-fit model (LG) estimated by ProtTest3 v3.4.2 (Darriba et al., 2011). FigTree v1.4.3^2^ and Adobe Illustrator CC 2017 were used to visualize and annotate the phylogenetic tree.

### Collinearity and Chromosomal Distribution of *OBP* Genes

We mapped the 44 *OBP* genes of *B. mori* (Gong D. P. et al., 2009) to the chromosomes in the newest version of the *B. mori* genome (Kawamoto et al., 2019) and rebuilt their gene structure by GMAP (Wu and Watanabe, 2005). However, only 43 *OBP* genes were successfully mapped: *BmorOBP24* is a pseudogene that was discarded. Subsequently, the best reciprocal BLAST hit was used to identify the orthologous *OBP* gene pairs in *C. pomonella* and *B. mori* genomes. MapGene2Chrom web v2^3^ was used to draw the distribution map of *OBP* genes on the chromosomes of both species. Orthologous gene pairs or blocks were linked by lines.

### Molecular Evolutionary Analysis

To estimate whether natural selection acted on the evolution of *OBP* genes in *C. pomonella*, we inferred the ratio of the normalized non-synonymous rate (*d*_N_) to the synonymous rate (*d*_S_) of nucleotide substitutions (ω = *d*_N_/*d*_S_) by a maximum likelihood method using the Codeml program in PAML v4.9b (Yang, 2007), with ω > 1, ω = 1, ω < 1 indicating positive selection, neutral evolution, and purifying selection, respectively. We first aligned the protein sequences for each analysis in MAFFT v7 (Katoh et al., 2002), then these protein alignments were converted to CDS alignments by the PAL2NAL program^4^. Subsequently, the protein alignments were trimmed by trimAl v1.2 (Capella-Gutierrez et al., 2009) and were used in MEGA6 (Tamura et al., 2013) to build Neighbor-Joining (NJ) trees with the Jones-Taylor-Thornton (JTT) model and 1,000 bootstrap replications.

We used the site model for each group of *OBP* orthologous/paralogous genes clustered by the phylogenetic tree to test which genes or sites might have evolved under positive selection. In this site model, we performed a test of heterogeneity across sites by comparing the M0 and M3 models with *K* = 3 categories. Another test of positive selection on sites involved fitting a beta distribution of ω values across sites by comparing M7 and M8 models. Considering the evolutionary specificity of *GOBP* and *PBP* genes in lepidopteran insects (Yasukochi et al., 2018), we used the branch-site model to test the genes as well as their amino acid sites that evolved under positive selection in nine lepidopteran insects including *B. mori*, *C. pomonella*, *D. plexippus*, *Heliconius melpomene*, *M. sexta*, *Operophtera brumata*, *Papilio xuthus*, *P. xylostella*, and *S. litura*. In the phylogenetic tree of each gene (*GOBP1*, *GOBP2*, *PBPA*, *PBPC*, and *PBPD*, but not *PBPB* due to lack of gene numbers), we labeled the branch composed of genes from *C. pomonella* as the foreground branch and the remaining branches as background branches to test positive selection in *C. pomonella GOBP* and *PBP* genes. We compared model A (the alternative model), in which some sites on the foreground branch were allowed to change to a value of ω > 1, with the null model of neutral evolution.

The likelihood ratio tests (LRTs) statistic (2ΔL), which approximates a χ^2^ distribution, was used for comparisons between models, and significant results were determined using χ^2^-tests. If the LRT was significant, Bayes empirical Bayes (BEB) was used to identify sites of positive selection. The sites with posterior probabilities (PPs) of ≥ 0.95 were considered positively selected, thus they were defined as positively selected sites (PSS).

### Homology Modeling and Molecular Docking

To further understand the functional significance of the identified positively selected sites, we labeled them on protein sequences and tertiary structures. The amino acid sequences of *CpomGOBP1* gene which evolved under positive selection in *C. pomonella* were submitted to the SWWISS-MODEL Server^5^ to predict and refine 3D structures. The best template was BmorGOBP2 (PDB ID: 2WCK), which has 53.57% identity with *CpomGOBP1*. Subsequently, we used the SAVES server^6^ and RAMPAGE server to estimate the quality of the predicted 3D structure. SAVES assesses the quality of protein 3D structure based on the PROCHECK (Laskowski et al., 1993), ERRAT (Colovos and Yeates, 1993), and VERIFY 3D (Lüthy et al., 1992) program. RAMPAGE assesses the quality based on the Ramachandran plot (Wang et al., 2016). The generated model structures were rendered and visualized using Visual Molecular Dynamics (VMD) v1.9.3 (Humphrey et al., 1996).

We collected 48 odorant molecules including pheromones and host plant volatiles by mining literatures (Bengtsson et al., 2001; Ansebo et al., 2004; Bengtsson et al., 2014; Knight et al., 2019). Then, docking was carried out by AutoDock Vina (Trott and Olson, 2010). The binding patterns between *CpomGOBP1* and the odorant molecule was visualized using VMD (Humphrey et al., 1996). The key binding site analysis was performed using LigPlus (Wallace et al., 1995).

### Expression Profiling of 40 *CpomOBPs*

We calculated the expression levels of 40 *CpomOBPs* in several sensory tissues based on transcriptome data. The sensory tissues were collected from female and male adults, including the antennae, head, leg, wing, labial palp, each sample with three biological replicates. The paired-end clean reads were mapped to the *C. pomonella* genome using HISAT2 (Kim et al., 2015). The FPKM (Fragments Per Kilobase of transcript per Million mapped reads) was calculated by StringTie software (Pertea et al., 2016). The heatmap.2 function of R package “gplots” to draw the heatmap of expression profiling based on the FPKM values.

## Results

### Identification of *OBP* Genes in *C. pomonella*

**FIGURE 1:**
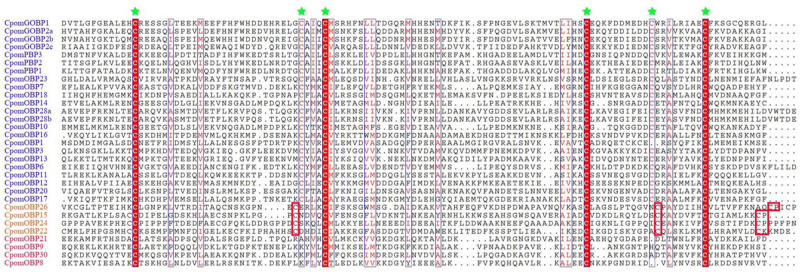
Amino acid alignment of various *C. pomonella OBP* family members. The alignment was performed by MAFFT v7, aligned sequences were depicted with ESPript 3.0 server (http://espript.ibcp.fr/ESPript/cgi-bin/ESPript.cgi). Highly conserved cysteine residues are marked by green stars. The gene IDs of classic, plus-C, and minus-C *OBPs* are shown in blue, orange and red color, respectively. Only the cysteines which are conserved in all of alignment sequences are highlighted in red block. Seven classic and two atypical *OBPs* are not shown in this figure.

A total of 40 *OBP* genes were identified in the *C. pomonella* genome. Complete CDS were determined by cross-checking with transcriptome assemblies. All gene information including gene names, CDS, amino acid sequences, chromosomes, and gene lengths and classification are provided in Supplementary Table 1. We compared the 40 *CpomOBPs* with those previous reported (Garczynski et al., 2013) and renamed them, and those that were not well matched were renamed by the sequence length. The amino acid sequences range from 133 to 339 amino acids (Supplementary Table 1). Multiple sequence alignments show that most have six typical conserved cysteine residues (Figure 1 and Supplementary Table 1). Based on the number and location of the conserved cysteines, the 40 *CpomOBPs* were classified into four subfamilies, including classic, minus-C, plus-C, and atypical. In total, 30 *CpomOBPs* belong to the classic subfamily, which had six typical conserved cysteine residues. Four *CpomOBPs* contained more cysteines than the classic *OBPs*, and were classified into the plus-C subfamily. Four *CpomOBPs* belong to the minus-C subfamily, which had fewer than six cysteines. The remaining two *CpomOBPs*, which exhibited none of the above characteristics, were classified into the atypical subfamily.

### Estimation of Gene Gains and Losses

**FIGURE 2:**
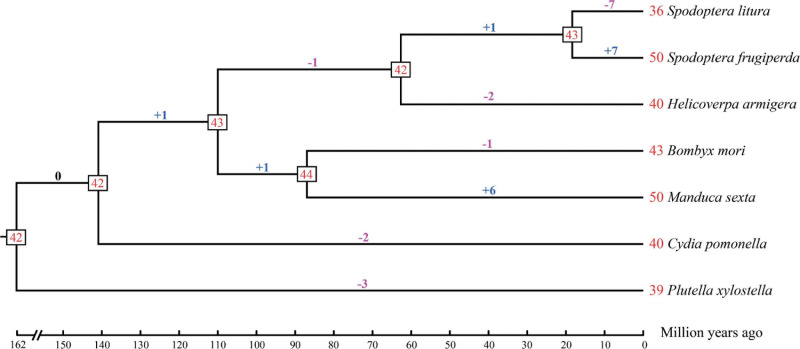
Estimation of *OBP* gene gains and losses during the evolution of seven moth species. The phylogenetic tree with estimated divergence times (million year ago) was inferred by MCMCTree in PAML v4.9b. The numbers at the tree termini are the numbers of genes in each species, which we found in the literature: *Spodoptera litura*, *Spodoptera frugiperda*, *Helicoverpa armigera*, *Manduca sexta*, and *Plutella xylostella*, the numbers of *OBP* genes in *Cydia pomonella* were identified in this study, while the number of *OBP* genes in *Bombyx mori* was corrected by this study (see section “Materials and Methods”). The numbers at the tree nodes are the numbers of genes in their most recent common ancestors. The numbers of gene gains and losses are shown above the branches, where the symbol “+” represents gene gains while “-” represents gene losses. The numbers before each species represent the *OBP* genes of the specific moth.

A statistical gene birth and death analysis for *OBP* genes from seven moth species (*S. litura*, *S. frugiperda*, *H. armigera*, *B. mori*, *M. sexta*, *C. pomonella*, and *P. xylostella*) was performed by CAFÉ (Figure 2). Forty-two *OBP* genes were inferred in the common ancestor node of moth species considered in this study at 162 Mya. The gene gains and losses range from -1 (lost one gene) to + 1 (gained one gene) between the adjacent ancestor nodes. However, different species have various gene gains or losses ranging from 1 to 7 compared with their adjacent ancestors. For example, gene gains occurred in *S. frugiperda* (+ 7) and *M. sexta* (+ 6), while gene losses occurred in *S. litura* (-7), *H. armigera* (-2), *B. mori* (-1), *C. pomonella* (-2), and *P. xylostella* (-3) compared with their adjacent ancestors. Our results suggested that the gains and losses of *OBP* genes may be associated with functional divergence which results from adaptation.

### Phylogenetic Analysis of *OBP* Genes

**FIGURE 3:**
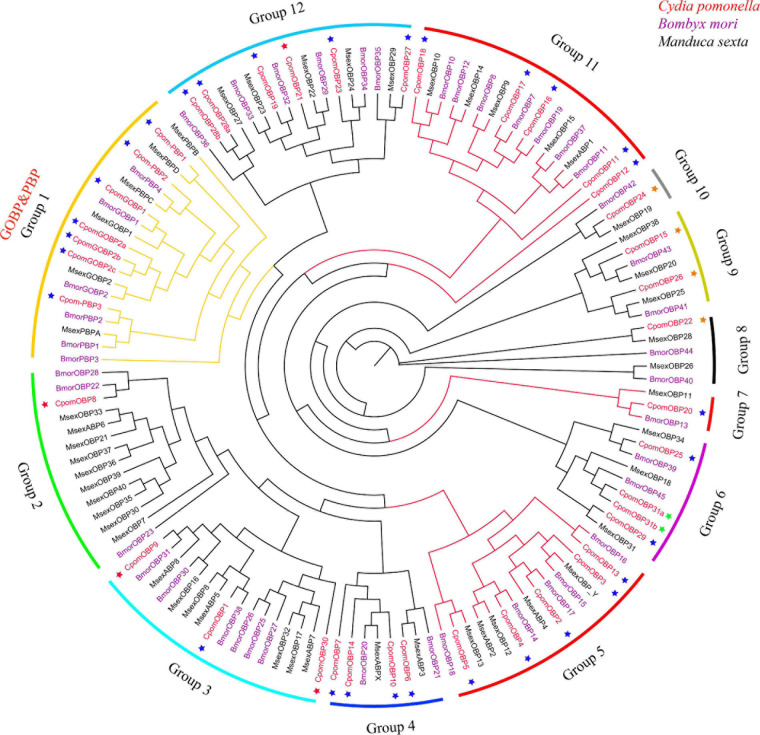
The phylogenetic tree of the *OBP* gene family in lepidopteran insects. *C. pomonella OBPs* are classified as Classic, Minus-C, Plus-C, and Atypical, which are represented by blue, red, orange, and green stars, respectively. The GOBP/PBP genes are shown in orange.

The phylogenetic tree was inferred using a total of 133 amino acid sequences of *OBP* genes, including 40 *OBPs* from *C. pomonella*, 43 *OBPs* from *B. mori*, and 50 *OBPs* from *M. sexta* (Figure 3). We classified these *OBP* genes into 12 groups (Groups 1–12) according to the clusters in the phylogenetic tree. Eleven of them were orthologous groups shared among these three species with nearly 1:1 orthologous genes in each group from each species, except that Group 2 has a lineage-specific expansion in *M. sexta*. The GOBP/PBP subfamily is a specific cluster in lepidopteran species (Vogt et al., 2015; Yasukochi et al., 2018), consisting of six typical tandem genes including *GOBP1*, *GOBP2*, and *PBPA*-*PBPD*. In our study, Group 1 was the conserved GOBP/PBP cluster, including six genes from *B. mori* (*GOBP1*, *GOBP2*, *PBPA-1*, *PBPA-2*, *PBPC*, and *PBPD*), six genes from *M. sexta* (*GOBP1*, *GOBP2*, *PBPA*, *PBPB*, *PBPC*, and *PBPD*), and seven genes from *C. pomonella* (*GOBP1*, *GOBP2a*, *GOBP2b*, *GOBP2c*, *PBP3*, *PBP2*, and *PBP1*). The *PBPB* gene was lost in *C. pomonella*, while the *GOBP2* gene is duplicated twice, which suggested that *GOBP2* may be under positive selection. In general, the *OBP* gene family is evolutionarily conserved in Lepidoptera insects.

### Collinearity and Chromosomal Distribution of *OBP* Genes

**FIGURE 4:**
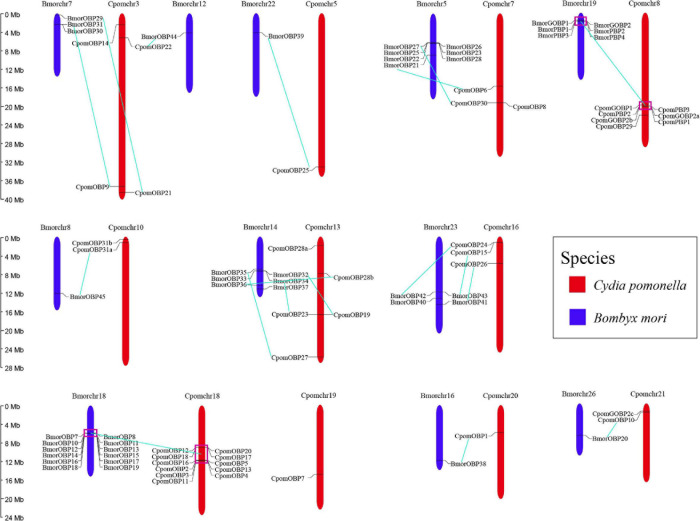
Chromosomal distribution of *OBP* genes in *C. pomonella* (red) and *B. mori* (blue). Cyan solid lines represent the correspondence between *C. pomonella* and *B. mori OBP* genes.

All 40 *OBP* genes were located on 11 *C. pomonella* chromosomes (Figure 4). These genes are organized into two major clusters on chromosomes 18 and 8, while the other chromosomes contain scattered and few *OBP* genes. The largest cluster contains 11 tandem *OBP* genes on chromosome 18, accounting for 27.5% of the total number of *OBP* genes. These genes have a collinearity block in chromosome 18 of *B. mori* that contains 12 tandem *OBP* genes. Furthermore, this collinearity block of *OBP* genes was clustered into Groups 5, 7, and 11 in the phylogenetic tree, which belong to antennal binding protein I (ABPI) and antennal binding protein II (ABPII) (Figure 3) (Gong D. P. et al., 2009). Another big cluster on chromosome 8 contains seven *OBP* genes, which account for 17.5% of the total number of *OBP* genes. Six of them were in a tandem *GOBP*/*PBP* gene cluster, including *GOBP1*, *GOBP2a*, *PBP3*, *PBP2*, and *PBP1*. There was also a tandem *GOBP*/*PBP* gene cluster on chromosome 19 in *B. mori*. There was a collinearity block of the *GOBP*/*PBP* gene cluster between *C. pomonella* and *B. mori*, and they were clustered into Group 1 in the phylogenetic tree (Figure 3). Seven *OBP* genes in *C. pomonella* have no collinearity compared with *B. mori*.

### Tests of Selective Pressures on Lepidopteran *OBP* Genes

We selected eight clades (groups) from the phylogeny to test whether some orthologous/paralogous *OBP* genes of three moths (including *B. mori*, *C. pomonella*, and *M. sexta*) evolved under positive selection. Selected groups included Groups 2–6, 9, 11, and 12 (Table 1). Groups 7–8 and 10 were excluded since they had too few genes. Group 1 was later tested independently using the branch-site model.

**TABLE 1:** Tests of positive selection on the orthologous/paralogous *OBP* genes of moths by site model.

**Clade**	**n**	***d*_N_/*d*_S_**	**2△l**	
			**M0 vs. M3**	**M7 vs. M8**
Group2	14	0.15846	55.305518** (*P* = 0)	14.265552 (*P* = 0.0008)**
Group3	16	0.11555	52.788572** (*P* = 0)	0.001414 (*P* = 0.9993)
Group4	8	0.04517	0 (*P* = 1)	7.520000 (*P* = 0.9996)
Group5	15	0.00687	135.091616** (*P* = 0)	0.001886 (*P* = 0.9991)
Group6	9	0.01691	0 (*P* = 1)	0.001218 (*P* = 0.9994)
Group9	7	0.00547	16.037306** (*P* = 0.0030)	3.500000 (*P* = 0.9998)
Group11	17	0.02222	13.897966** (*P* = 0.0076)	7.020000 (*P* = 0.9996)
Group12	17	0.01384	0 (*P* = 1)	0.001418 (*P* = 0.9993)
**Clade**	**Parameter estimated under M8 model**			**Positively selected sites (PSSs) from Bayes empirical Bayes (BEB) analysis**
Group2	p0 = 0.98223, *p* = 3.14309, *q* = 15.84241, p1 = 0.01777, ω = 3.42058			5T (0.934)

n, Number of genes tested; d_N_/d_S_, Estimated under M0; 2△l, Likelihood ratio test.

**Significant within the 1% interval after Bonferroni correction.

According to tests of the one-ratio model (M0), which assumes a single ω for all amino acid sites, the ω values of eight clades ranged from 0.00547 to 0.15846 (Table 1), suggesting the existence of strong purifying selection. However, the comparison between models M0 and M3 (discrete) provided strong evidence of variation in selective pressures at different amino acid sites in Groups 2–3, 5, 9, and 11 (*P* < 0.01, Table 1), indicating that purifying selection has been relaxed at some amino acid sites. We further compared models M7 and M8 for clades showing 0.5 < *d*_S_ < 1 to investigate whether some amino acid sites actually evolved under positive selection. Only Group 2 presented evidence of positive selection (*P* = 0.0008) with one positively selected site (PSS). However, the Bayes empirical Bayes (BEB) analysis showed that the PSS only had a 93.4% posterior probabilities (PPs), which is not statistically significant.

**FIGURE 5:**
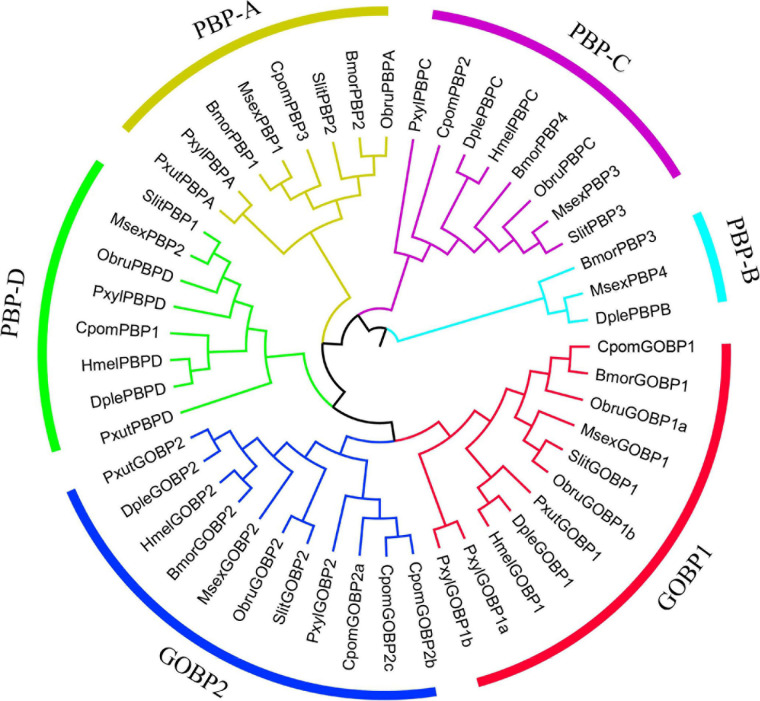
Phylogenetic relationships of *GOBP* and *PBP* genes in lepidopteran insects. The maximum likelihood tree was constructed based on the 49 *GOBP* and *PBP* genes from nine lepidopteran species. The amino acid sequences were aligned by MAFFT v7 with default parameters, and then the alignments were trimmed by trimAl with the parameter “-automated1.” (Bmor: *Bombyx mori*, Cpom: *Cydia pomonella*, Dple: *Danaus plexippus*, Hmel: *Heliconius melpomene*, Msex: *Manduca sexta*, Obru: *Operophtera brumata*, Pxut: *Papilio xuthus*, Pxyl: *Plutella xylostella*, Slit: *Spodoptera litura*). MEGA v6 was used to construct a Neighbor-Joining tree with the Jones-Taylor-Thornton (JTT) model and 1,000 bootstrap replications. FigTree v1.4.3 (http://tree.bio.ed.ac.uk/software/figtree/) and Adobe Illustrator CC 2017 were used to visualize and annotate the phylogenetic tree.

We used the branch-site model to test the positive selection in each codon for different gene clades of GOBPs and PBPs from nine species (*B. mori*, *C. pomonella*, *D. plexippus*, *Heliconius melpomene*, *M. sexta*, *Operophtera brumata*, *Papilio xuthus*, *P. xylostella*, and *S. litura*) (Figure 5). Only GOBP1 was identified as being under positive selection for *C. pomonella* after the likelihood ratio test (*P* = 0.0318). We further used the BEB approach to detect the positive sites in GOBP1, which showed that sites 41S and 43G were significant signs of positive selection with PPs of 0.994 and 0.972, respectively (Table 2).

**TABLE 2:** Tests of positive selection on Lepidopteran *GOBP* and *PBP* genes by branch-site model (Branch labels referring to Figure 5).

**Branch-site model**	**H0 lnL Vs. H1 lnL**	**df**	2**△l and *P*-value**	**Parameter Estimated under H1**	**Positively Selected Sites (PSSs)**
GOBP1	–3159.24	1	4.61	p_0_ = 0.809, p_1_ = 0.140	**41S** 43G
	–3156.94		*P* = 0.0318*	p_2a_ = 0.043, p_2b_ = 0.007	
				ω_1_ = 1.000, ω_2_ = 533.665	
GOBP2	–3897.96	1	2.89	p_0_ = 0.861, p_1_ = 0.107	N/A
	–3896.51		*P* = 0.0890	p_2a_ = 0.028, p_2b_ = 0.004	
				ω_1_ = 1.000, ω_2_ = 999.000	
PBP3	–3313.48	1	0.18	p_0_ = 0.639, p_1_ = 0.104	26Q 46E 112D 115T **148K** 149G 150M 153S
	–3313.39		*P* = 0.6755	p_2a_ = 0.221, p_2b_ = 0.036	
				ω_1_ = 1.000, ω_2_ = 1.269	
PBP2	–3414.50	1	0	p_0_ = 0.759, p_1_ = 0.191	N/A
	–3414.50		*P* = 1.0000	p_2a_ = 0.040, p_2b_ = 0.010	
				ω_1_ = 1.000, ω_2_ = 1.000	
PBP1	–3254.95	1	0.41	p_0_ = 0.769, p_1_ = 0.197	N/A
	–3254.75		*P* = 0.5212	p_2a_ = 0.027, p_2b_ = 0.007	
				ω_1_ = 1.000, ω_2_ = 2.733	

*Significant within the 5% interval after Bonferroni correction.

**Significant within the 1% interval after Bonferroni correction.

*PSSs in bold show 99% posterior probability confidence.*

*N/A: No positively selected sites were detected.*

### Structural Links to Protein Function

**FIGURE 6:**
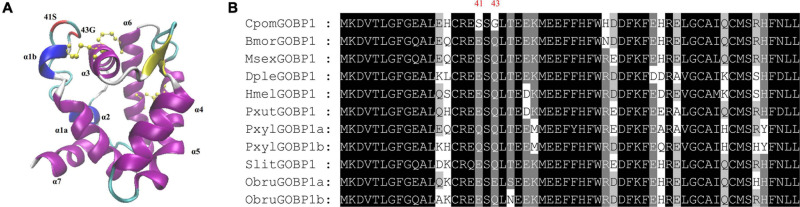
Structural homology model and positively selected sites of *C. Pomonella* GOBP1. **(A)** Structural homology model of *C. pomonella* GOBP1, positively selected sites are marked in red. **(B)** Multiple sequence alignments of GOBP1 genes from ten moth species.

To get additional insight into the functional significance of PSSs, we mapped the PSSs to the multiple sequence alignments of GOBP1 protein sequences from nine species, and labeled them on the structural homology model of *C. pomonella* GOBP1 (Figure 6A). Compared to the other eight species, the 41st amino acid Glutamic acid (E) was substituted by Serine (S), while the 43rd amino acid Glutamine (Q) was substituted by Glycine (G) (Figure 6B). The structural homology model of *C. pomonella* GOBP1 showed that both the 41st and 43rd amino acids were located in the loop near the first helix (Figure 6A).

**FIGURE 7:**
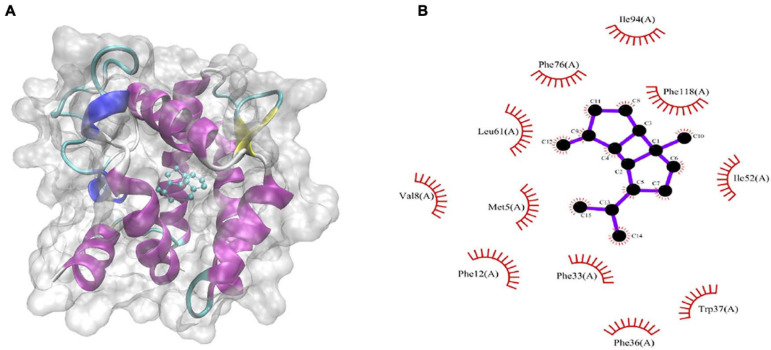
Molecular docking of *CpomGOBP1* with β-bourbonene. **(A)** β-bourbonene located in the binding cavity of *CpomGOBP1*. **(B)** Key amino acid residues that interact with β-bourbonene.

The binding energies that *CpomGOBP1* bind with 48 odorant molecules were assessed by AutoDock Vina (Trott and Olson, 2010). Among them, β-bourbonene had the lowest binding energy (-9.3 KJ/mol) with *CpomGOBP1* (Supplementary Table 2). β-bourbonene was located in the binding cavity composed of 11 hydrophobic amino acid residues, including Phe12, Phe33, Phe36, Phe76, Phe118, Ile52, Ile94, Val8, Trp37, Met5, and Leu61 (Figures 7A,B).

### Expression Profiling of 40 *CpomOBPs*

**FIGURE 8:**
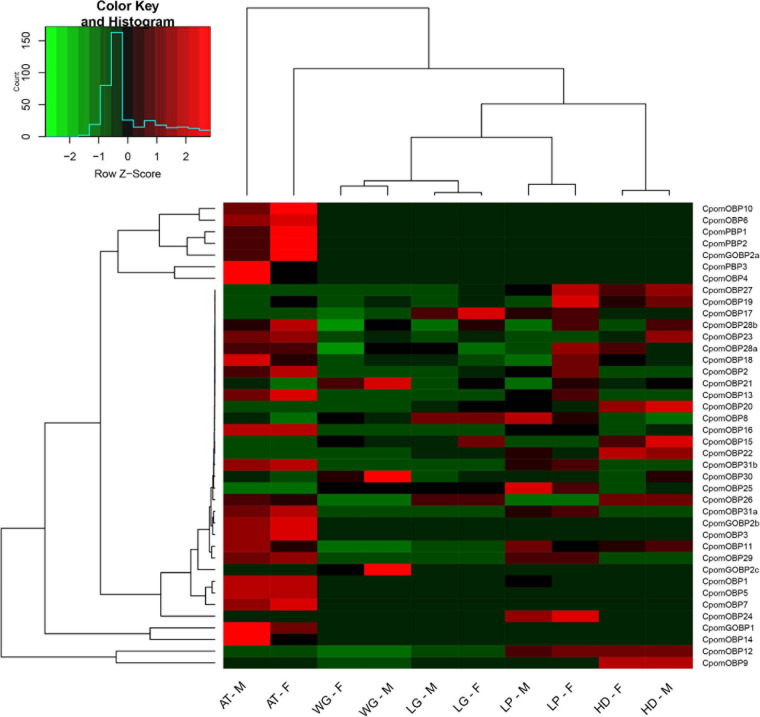
Expression profiling of 40 *CpomOBPs* in different tissues. F, female; M, male; AT, antenna; HD, head (antennae removed); WG, wing; LG, leg; LP, labial palp.

The expression profiling of all 40 *CpomOBPs* were assessed using FPKM values based on transcriptome data (Figure 8). The result showed that 31 *CpomOBPs* expressed (FPKM ≥ 10) in the antennae, head, leg, wing, and labial palp, except *CpomOBP23*, *CpomOBP2*, *CpomOBP18*, *CpomOBP28a*, *CpomOBP28b*, *CpomOBP19*, *CpomOBP21*, *CpomOBP27*, and *CpomOBP17*. There were 25 and 26 *CpomOBPs* that are expressed in the antennae of female and male adults, respectively, 22 of them were classic *OBPs*. The other enriched expression tissue is the labial palp, in which there are 27 and 25 *CpomOBPs* expressed in female and male adults. Three *CpomPBPs*, *CpomGOBP1*, *CpomGOBP2a*, and *CpomGOBP2b*, were mainly expressed in the antennae and labial palp. It is interesting to note that *CpomGOBP2c* was specifically expressed in wing.

## Discussion

In this study, we identified 40 *OBP* genes in *C. pomonella*, which is similar to several lepidopteran moth species e.g., 43 *OBPs* in *B. mori* (Gong D. P. et al., 2009), 39 *OBPs* in *P. xylostella* (Cai et al., 2020), 40 *OBPs* in *H. armigera* (Pearce et al., 2017), and 36 *OBPs* in *S. litura* (Cheng et al., 2017). However, *C. pomonella* has fewer *OBPs* than *M. sexta* and *S. frugiperda*, both of which have 50 *OBP* genes (Gouin et al., 2017). Variation in the numbers of *OBP* genes among moth species suggests that the evolution of *OBP* genes occurred during the speciation adaptation process and functional requirements for each species. A study in *B. mori* found that classic *OBPs* were dominant in the *OBP* gene family: *B. mori* has 29 classic *OBPs*, five plus-C *OBPs*, and eight minus-C *OBPs* (Gong D. P. et al., 2009). Similarly, in our study the 40 *OBP* genes of *C. pomonella* were classified as 30 classic *OBPs*, four plus-C *OBPs*, four minus-C *OBPs*, and two atypical *OBPs*. A previous study suggested that most of the classic *OBPs* and all ABPIIs are likely involved in chemoreception, since they show increased chemosensory tissue expression (Dippel et al., 2014). The fact that 75% of *OBPs* in *C. pomonella* are classic *OBPs* indicated that these genes are essential in recognizing host plants or pheromones such as sex pheromones. The result of expression profiling indicates that 22 classic *OBPs* were expressed in the antennae, which is similar to the finding in *Tribolium castaneum* (Dippel et al., 2014).

We used the CAFÉ software to estimate gene gains and losses, rather than directly comparing the number of *OBP* genes, because it considers a birth-and-death model in the evolutionary process (Han et al., 2013). In the most recent common ancestor of moths considered in this study, approximately 162 Mya inferred by two time frames adopted from TimeTree (see section “Materials and Methods”), 42 *OBP* genes were shared. There were no more than two expanded and contracted genes in each ancestor node, which indicated that speciation may not be driven by the evolution of *OBP* genes. The gene gains or losses of each species compared to their distant ancestors range from 1 to 7. According to this result, we suggest that the functional divergence of *OBP* genes occurred mainly after speciation, as a result of adapting to a new diversity of environments such as new host plants or pheromones. As a result, the *OBP* genes may be under positive selection. However, the variation of *OBPs* in moths is smaller than the odorant receptors (ORs) or gustatory receptors (GRs): the expanded or contracted genes of these two gene families is as high as 54 (Engsontia et al., 2014). In general, we found that *C. pomonella* lost two *OBP* genes compared to its closest ancestor.

To further explore which *OBP* genes have expanded or contracted in *C. pomonella*, we built a phylogenetic tree and performed collinearity analysis in chromosome location by comparing with related moth species. The results showed that *OBP* genes were conserved except the genes in Group 2, which contains many expanded genes in *M. sexta*. We also noticed some gene gains and losses in the conserved clade Group 1, composed of *GOBP* and *PBP* genes. The *GOBPs* and *PBPs* were a specific conserved subfamily in butterflies and moths, including GOBP1-2 and PBPA-D, which are in a tandem array with a fixed order in the same chromosome. These genes were thought to be involved in the recognition of volatile organic compounds (VOC) and sex pheromones of insects (Liu et al., 2020). However, recent studies showed some variations in this subfamily, including gene gains, losses, inversions, and translocations (Yasukochi et al., 2018). Although *GOBP1* and *GOBP2* were regarded as conserved in lepidopteran species (Vogt et al., 2015), some studies found that gene gains occurred in the *GOBP1* genes, such as duplication events of *GOBP1* in *P. xylostella* (Yasukochi et al., 2018) and *Operophtera brumata* (Yasukochi et al., 2018). In our study, we found a duplication event of a *GOBP2* gene that generated three *GOBP2* (*GOBP2a*, *GOBP2b*, and *GOBP2c*), two of which have been reported by Garczynski (Garczynski et al., 2013). *PBP* gene gains and losses occurred more commonly; most Lepidoptera have lost the *PBPB* gene (Yasukochi et al., 2018), while *PBPA* was expanded in *B. mori* (Gong D. P. et al., 2009). Similarly, we found that the *PBPB* gene was also lost in *C. pomonella*, which suggests that this gene may be undergoing a gene fusion event (Yasukochi et al., 2018).

Most *OBP* genes result from tandem duplications in insects, such as *Drosophila melanogaster* (Hekmat-Scafe et al., 2002) and *Anopheles gambiae* (Xu et al., 2003) in Diptera; *Tribolium castaneum* (Dippel et al., 2014) in Coleoptera; and *B. mori* (Gong D. P. et al., 2009) and *C. pomonella* (this study) in Lepidoptera. However, in earlier diverging ancestor orders including Hemiptera and Hymenoptera, there are fewer *OBP* genes without large tandem duplications (Vieira and Rozas, 2011), as in *Acyrthosiphon pisum* (Zhou et al., 2010) and *Bemisia tabaci* (Zeng et al., 2019) in Hemiptera; and *Apis mellifera* (Foret and Maleszka, 2006) and *Solenopsis invicta* (Pracana et al., 2017) in Hymenoptera. We also found a very consistent collinearity between *B. mori* and *C. pomonella*. These findings strongly suggest that the expansion of most *OBP* genes is caused by tandem duplications, and the tandem duplications of *OBP* genes in Lepidoptera occurred before speciation, indicating the existence of mainly purifying selection in moth *OBP* genes. In addition, the duplicated *CpomGOBP2c* gene is located in chromosome 21, instead of the GOBP/PBP gene cluster in chromosome 8, which indicates functional differentiation.

Some studies showed that single-point mutation of an amino acid could cause functional differentiation (Leary et al., 2012; Yang et al., 2017). Therefore, we further tested whether there are some positive sites in the *OBP* genes in *C. pomonella*. The results of evolutionary analysis by site model showed that most *OBP* genes evolved under purifying selection with ω ranging from 0.00547 to 0.15846 estimated by the M0 model. Similarly, most *OBP* genes in *B. mori* also evolved under purifying selection (Gong D. P. et al., 2009). The purifying selection of *OBP* genes is potentially due to functional constraints (Gong D. P. et al., 2009). However, among the *OBP* genes of lepidopteran species, the major function of *PBP*s is mainly to sense pheromones (Gong Y. et al., 2009), while *GOBP*s mainly sense the volatiles of host plants (Vogt et al., 2002). We assumed that *GOBP/PBP* genes may evolve under positive selection due to the vast diversity of sex pheromones and host volatiles. The results of the branch-site model on *GOBP/PBP* genes suggested that the *GOBP1* gene in *C. pomonella* evolved under positive selection. We detected two positively selected sites (41 S and 43 G) in *CpomGOBP1*, both located in the loop, close to the first disulfide bridge on helix 1. The mutations of these two amino acid residues may influence the fold shape of the binding cavity by modifying the disulfide bridge, which will cause functional differentiation (Sanchez-Gracia and Rozas, 2008). The docking result suggests that *CpomGOBP1* may have the ability to bind with β-bourbonene, however this must be functionally validated.

## Data Availability Statement

The original contributions presented in the study are included in the article/Supplementary Material, further inquiries can be directed to the corresponding author/s.

## Author Contributions

FW, WQ, and NY conceived, designed this study, and revised the manuscript. CH, XZ, DH, QW, RT, LX, WL, WW, BL, and YX collected the data and completed bioinformatics analysis. CH and XZ drafted the manuscript. All authors read and approved the final manuscript.

## Conflict of Interest

The authors declare that the research was conducted in the absence of any commercial or financial relationships that could be construed as a potential conflict of interest.

## Footnotes

1 http://espript.ibcp.fr/ESPript/cgi-bin/ESPript.cgi

2 http://tree.bio.ed.ac.uk/software/figtree/

3 http://mg2c.iask.in/mg2c_v2.1/

4 http://www.bork.embl.de/pal2nal/

5 https://swissmodel.expasy.org/

6 https://servicesn.mbi.ucla.edu/SAVES/
