# Supplementary material for: Comparative Genomics Provide Insights Into Function and Evolution of Odorant Binding Proteins in Cydia pomonella
Source: Front Physiol. 2021 Jul 7;12:690185. doi: 10.3389/fphys.2021.690185 (PMC8294088; doi:10.3389/fphys.2021.690185)
Supplement: Supplementary file 2 [file Table_2.doc]

Table S2 Binding energy of CpomGOBP1 with 48 odorant molecular

| Number | Ligand | CAS number | Structure | Binding Energy (KJ/mol) |
| --- | --- | --- | --- | --- |
| 1 | β-bourbonene | 5208-59-3 | 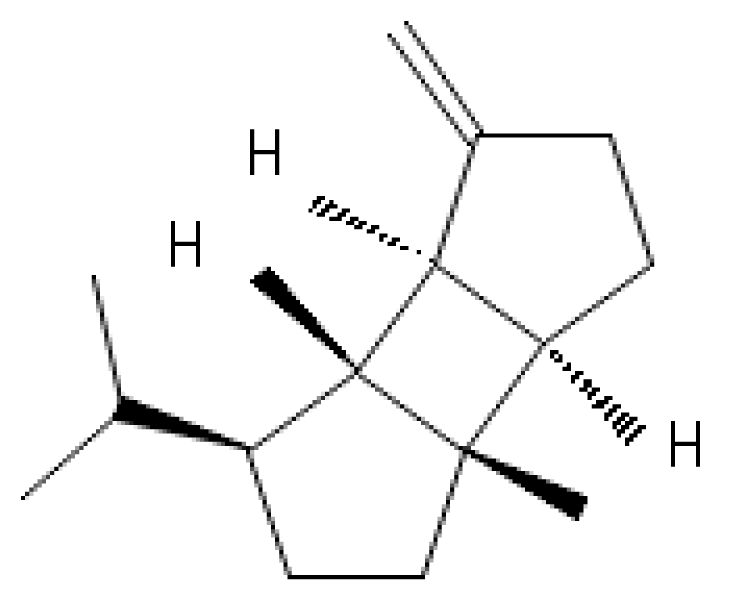 | -9.3 |
| 2 | Germacrene D | 23986-74-5 | 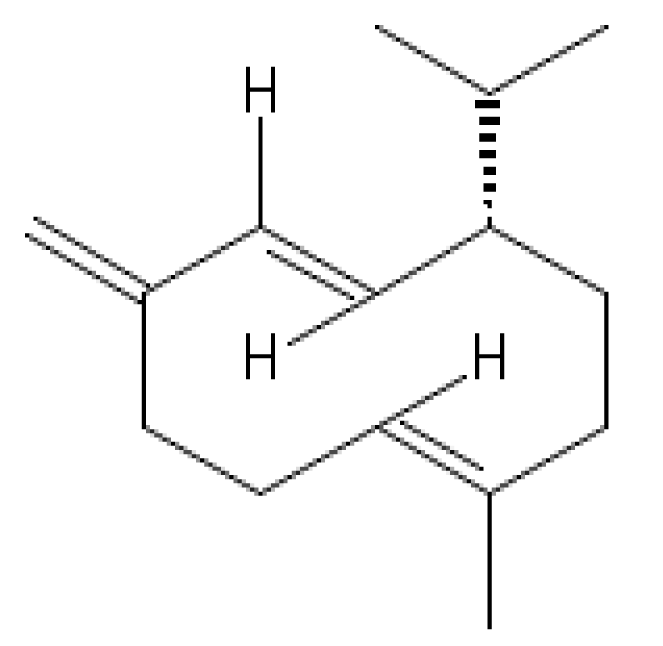 | -9.2 |
| 3 | beta-Caryophyllene | 87-44-5 | 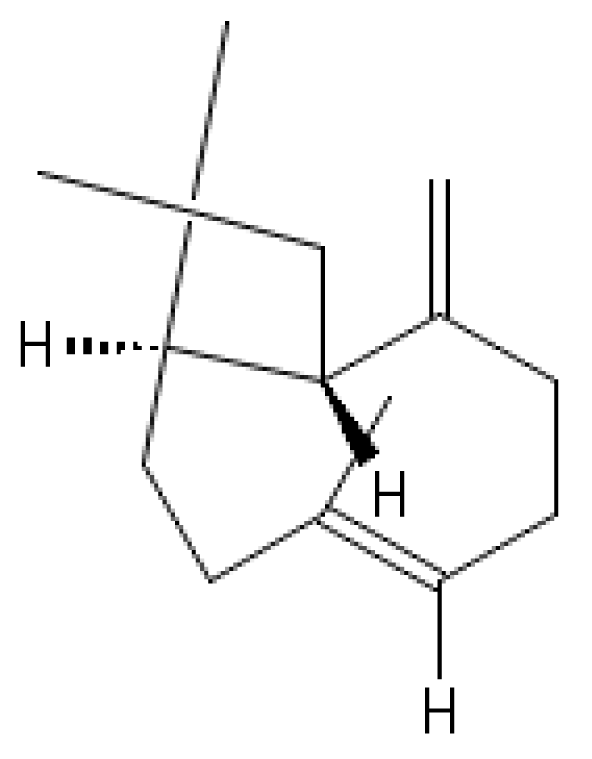 | -9.1 |
| 4 | (Z,E)-α-farnesene | 26560-14-5 | 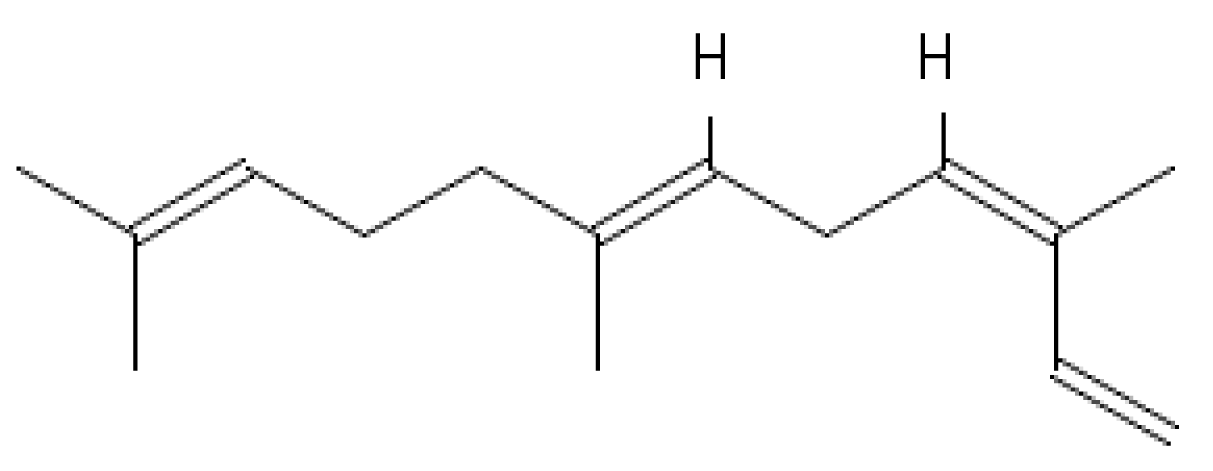 | -8.4 |
| 5 | (E,E)-α-Farnesene | ‎502-61-4 | 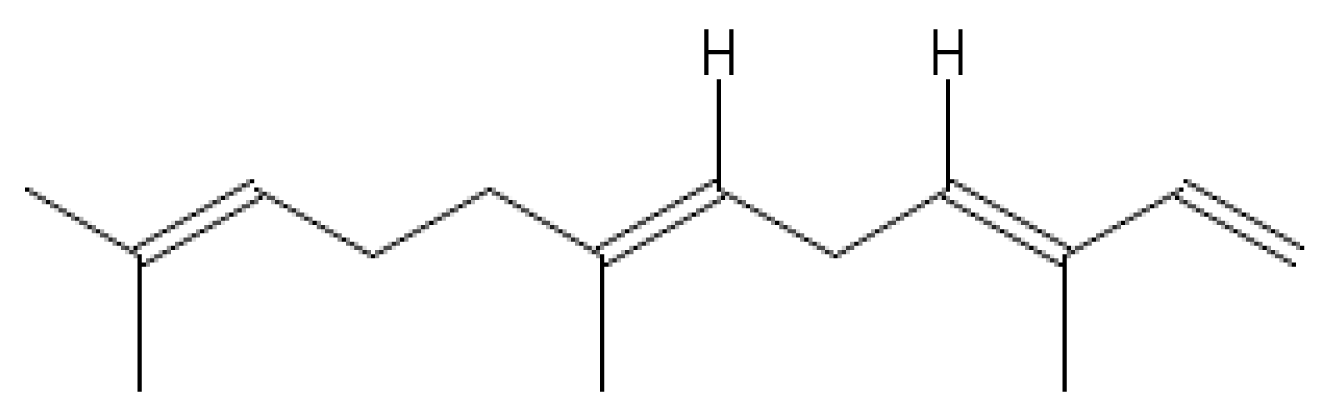 | -8.4 |
| 6 | (E)-β-Farnesene | 18794-84-8 | 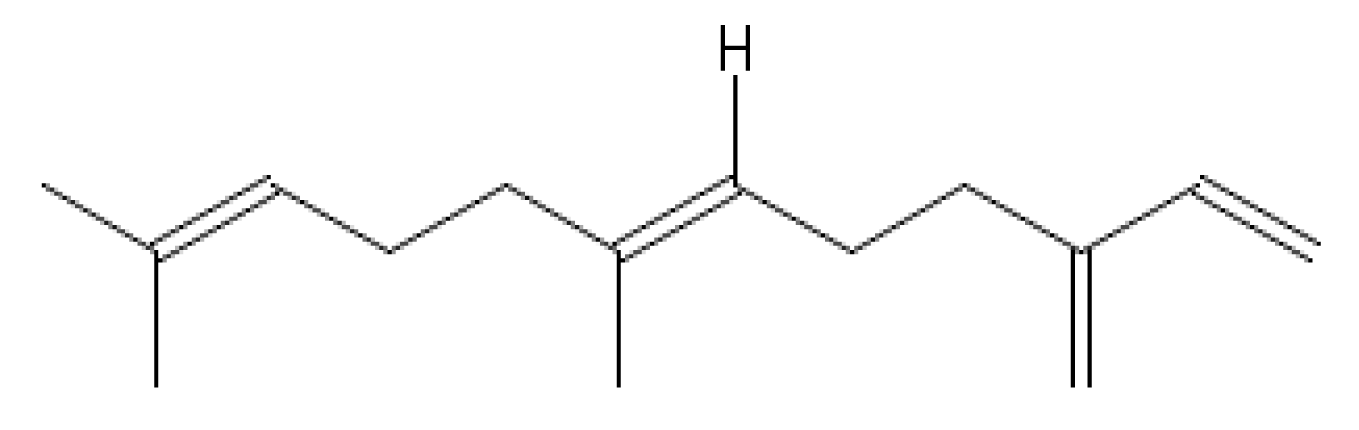 | -8.1 |
| 7 | (Z)3-Hexenyl benzoate | 25152-85-6 | 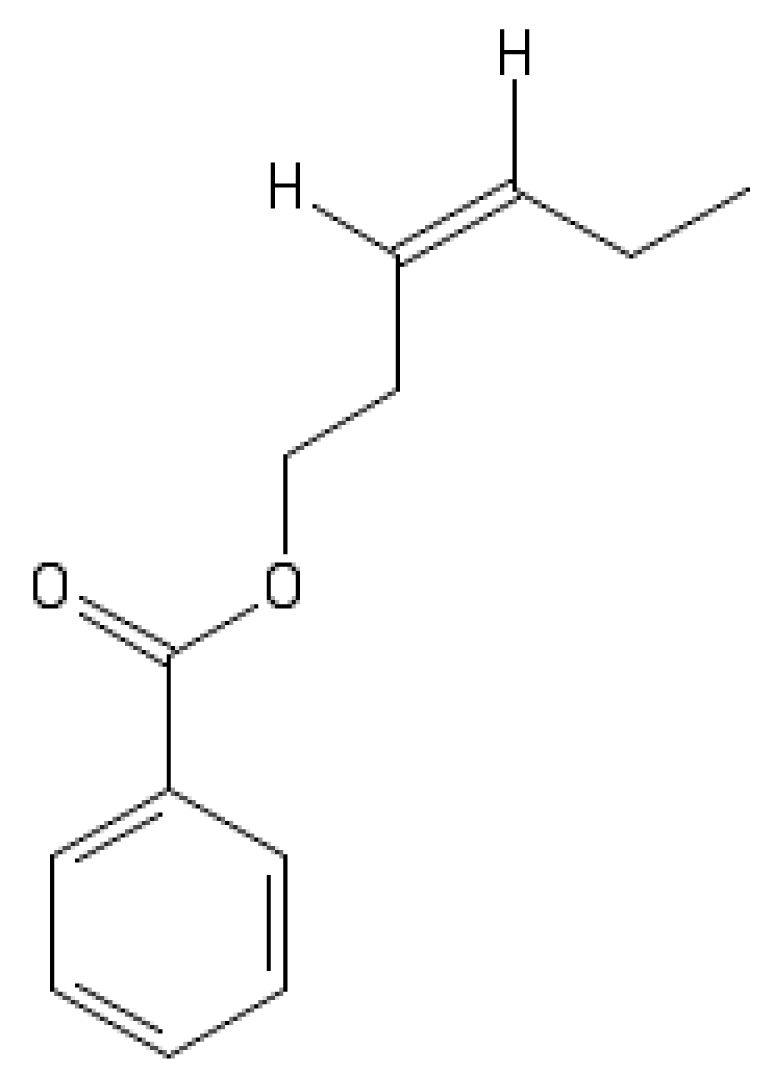 | -7.7 |
| 8 | (Z,E)-9,12-tetradecadienyl acetate | 30507-70-1 | 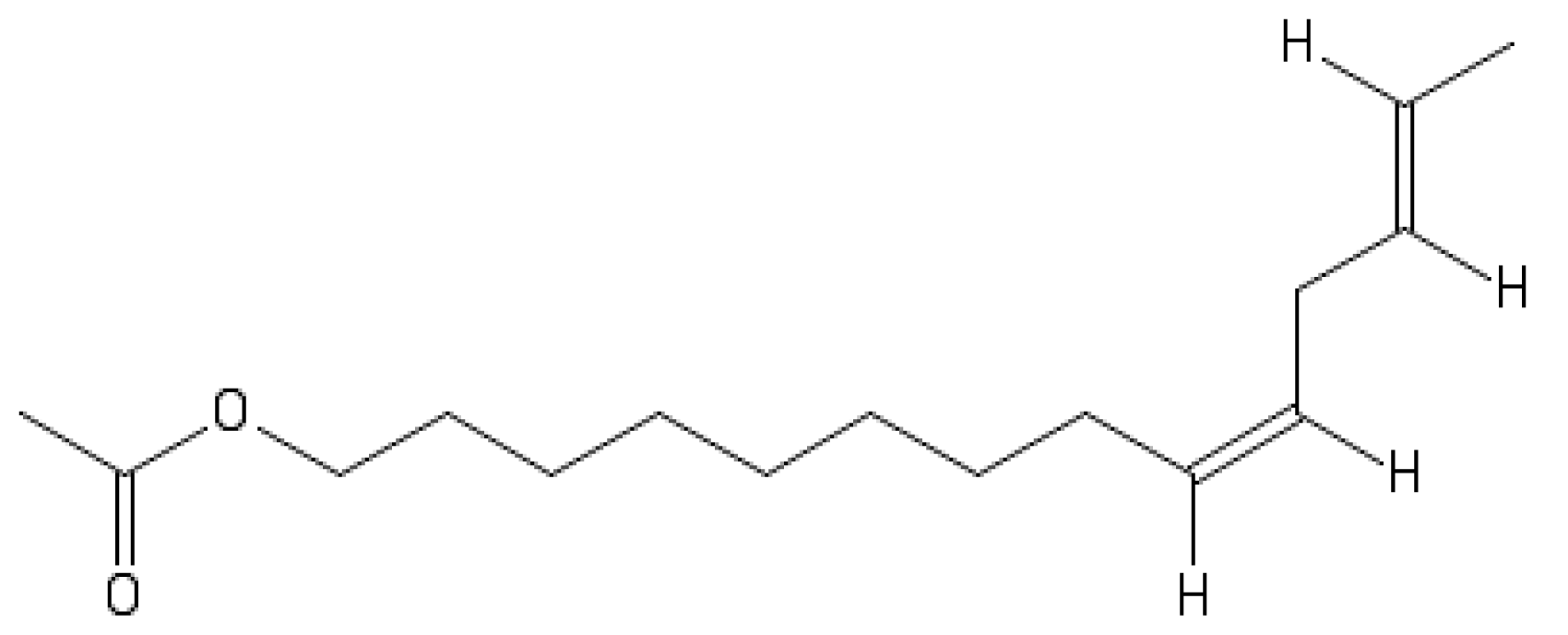 | -7.5 |
| 9 | 4,8-dimethyl-1,3,(E)7-nonatriene | 19945-61-0 | 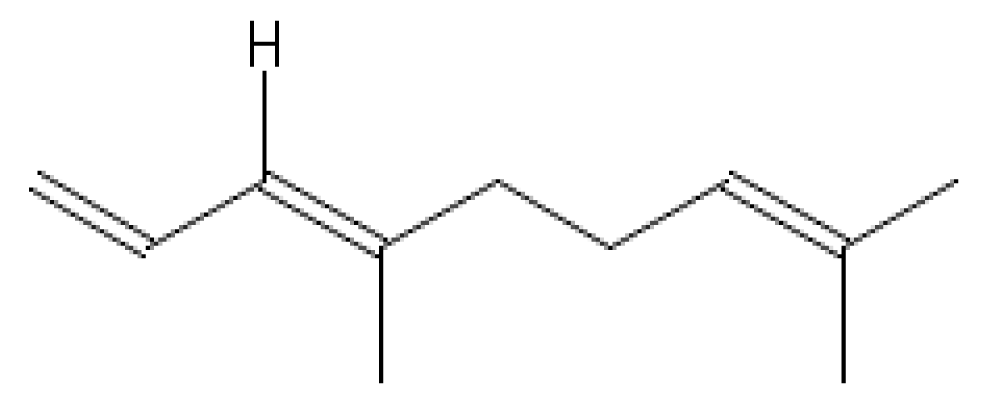 | -7.3 |
| 10 | (E,E)-8,10-Dodecadienol acetate | 53880-51-6 | 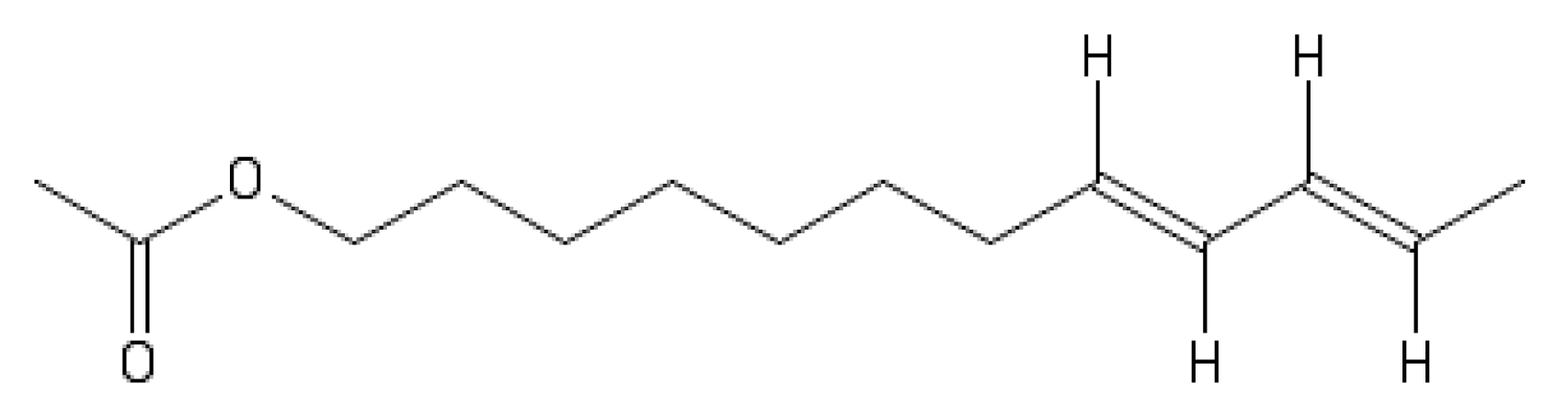 | -7.1 |
| 11 | (Z,E)-8,10-Dodecadienol | 33956-51-3 | 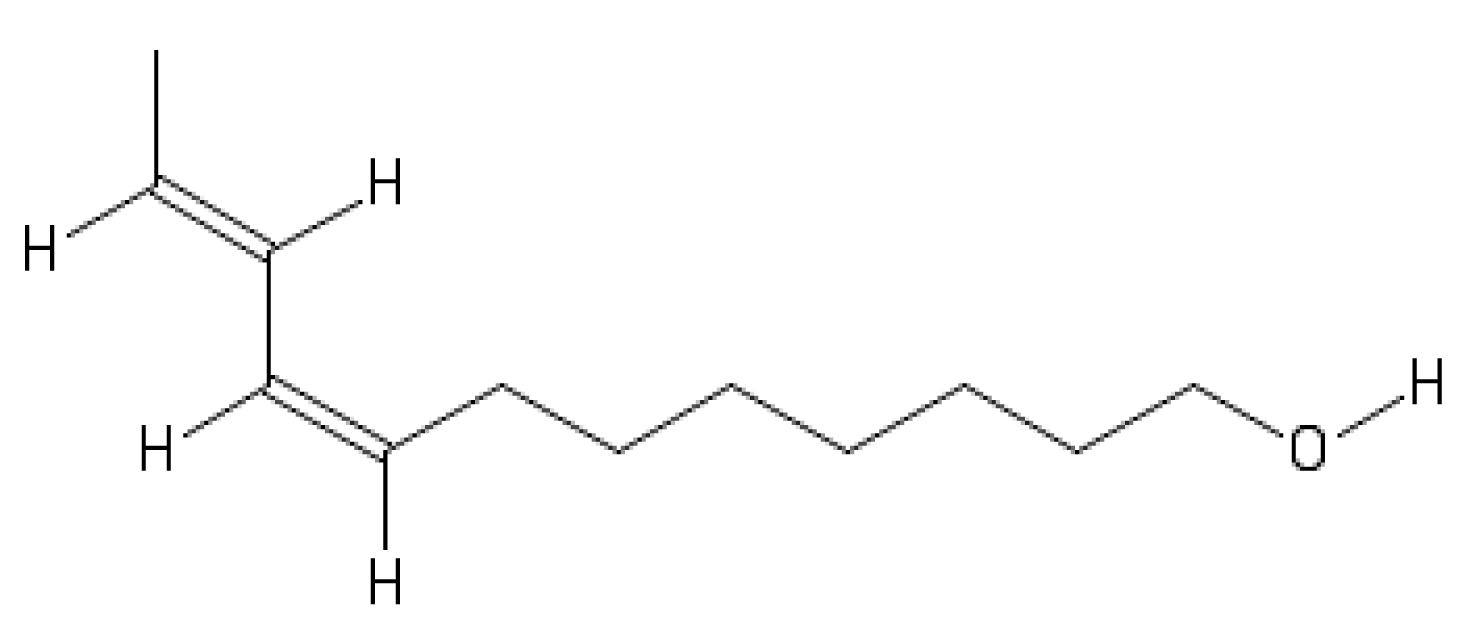 | -6.9 |
| 12 | (Z,Z)-8,10-Dodecadienol | 39616-21-2 | 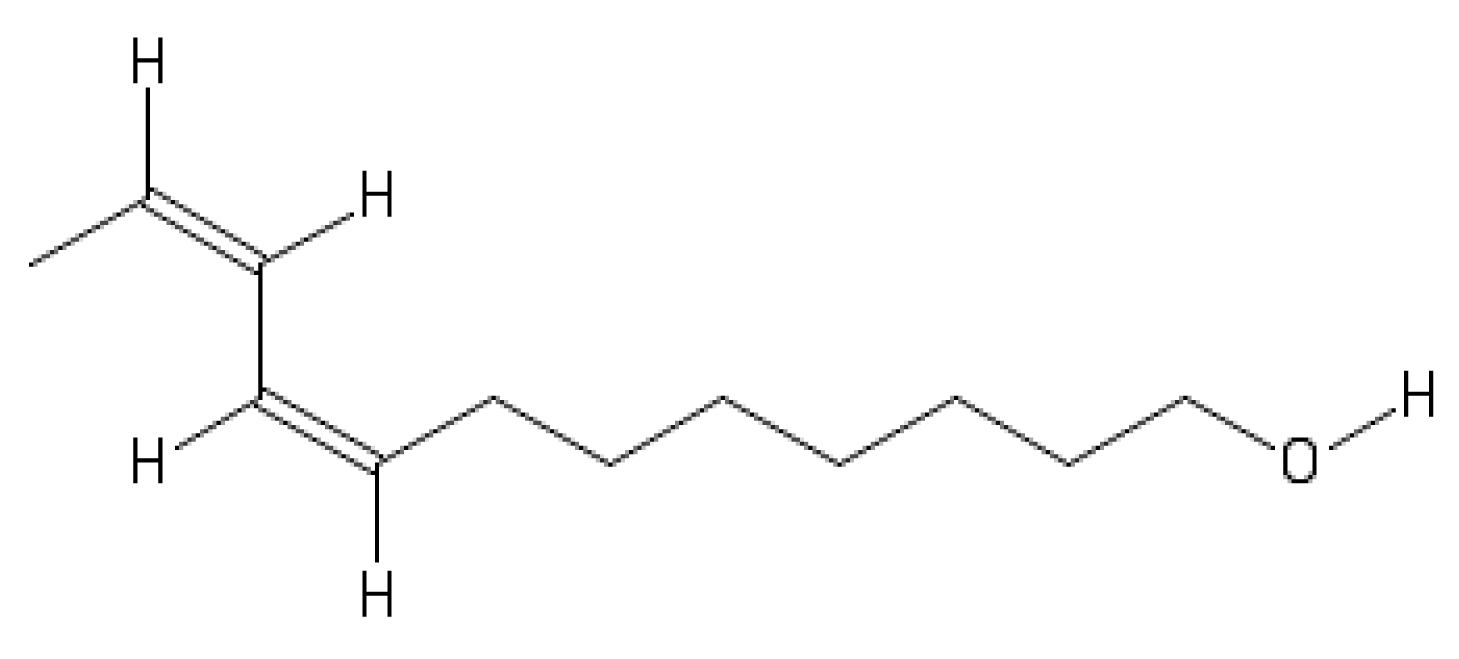 | -6.9 |
| 13 | (E)-β-ocimene | 3779-61-1 | 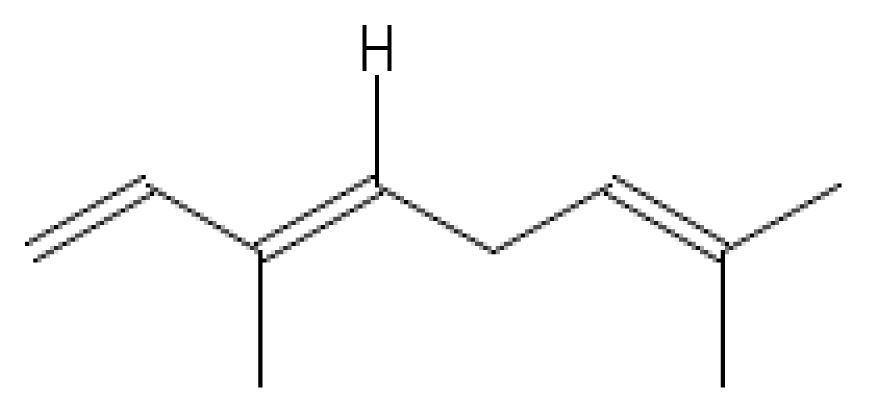 | -6.8 |
| 14 | (E,Z)-8,10-Dodecadienol | 33956-50-2 | 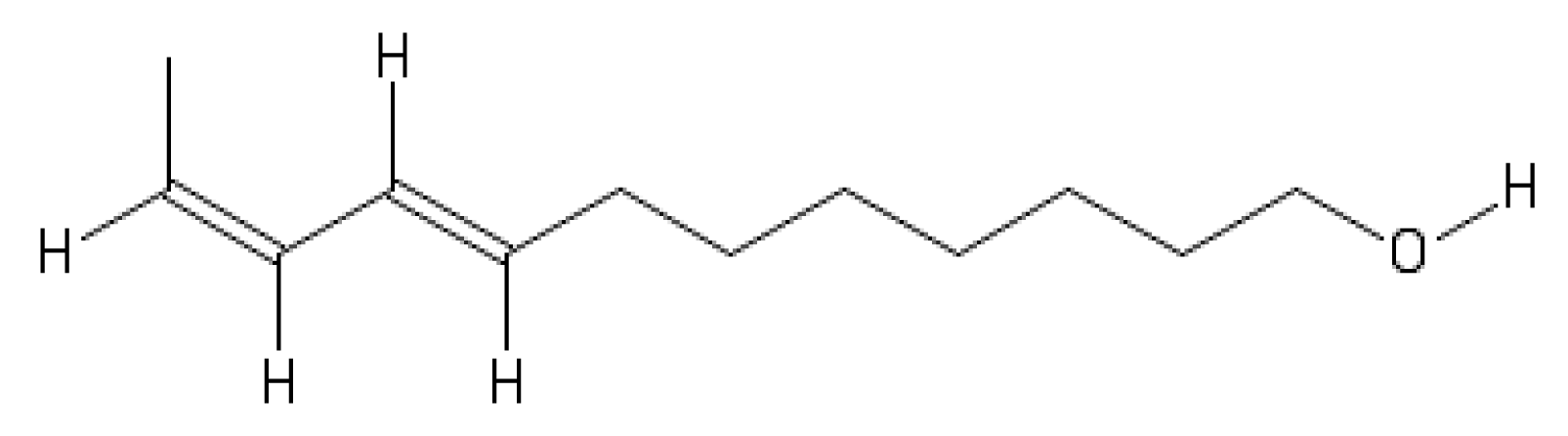 | -6.7 |
| 15 | Ethyl-(E,Z)-2,4-Decadienoate | 3025-30-7 | 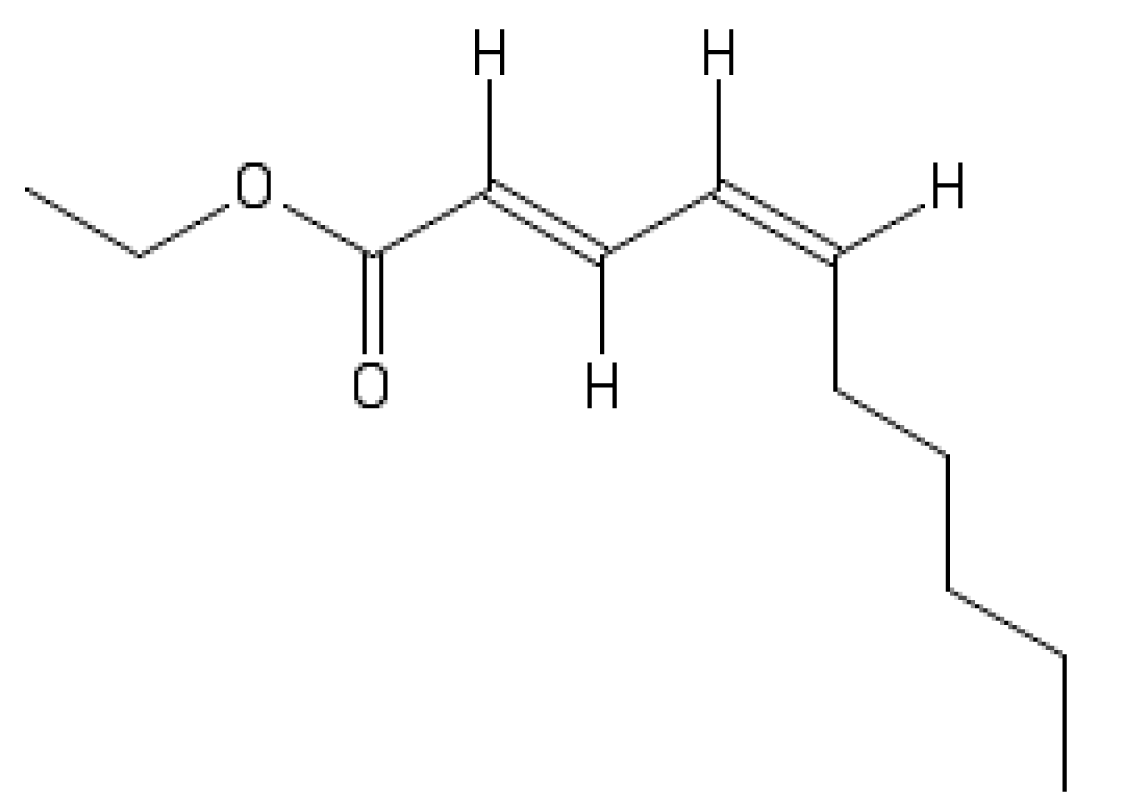 | -6.7 |
| 16 | (E,E)-8,10-Dodecadienol | 33956-49-9 | 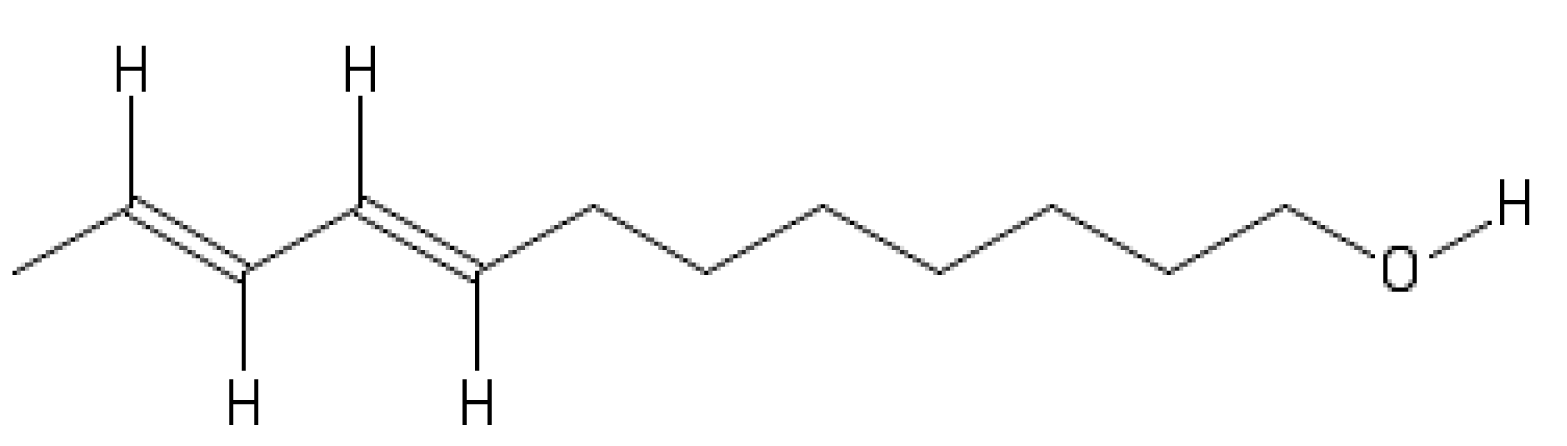 | -6.6 |
| 17 | (E)-9-Dodecenol | 35237-62-8 | 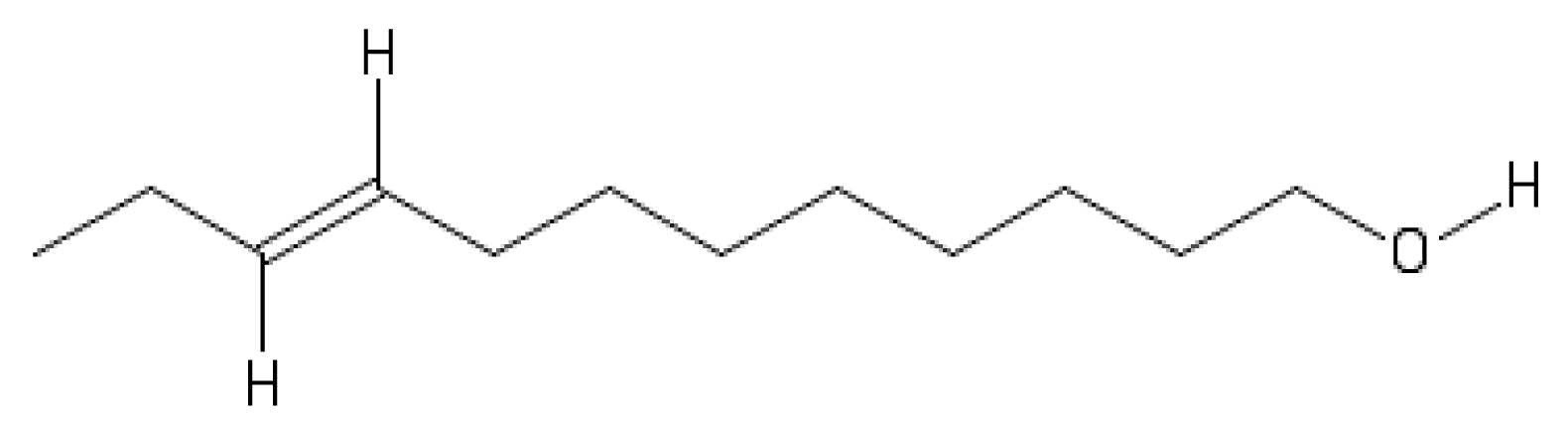 | -6.6 |
| 18 | (E)-10-Dodecenol | 35237-63-9 | 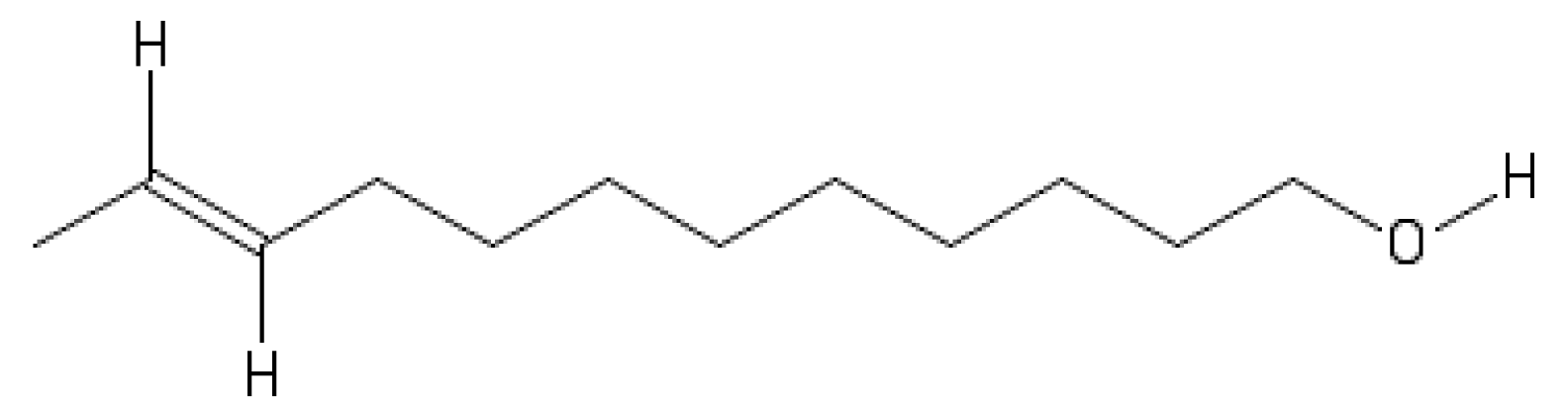 | -6.6 |
| 19 | E,E-2,4-dodecadienal | 21662-16-8 | 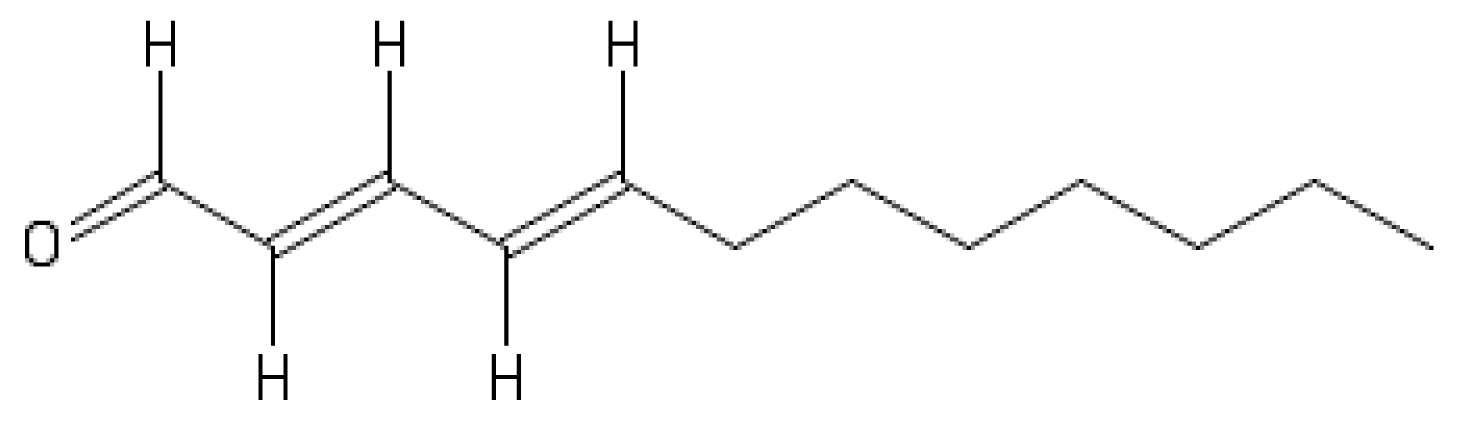 | -6.6 |
| 20 | (E)-8-Dodecenol | 42513-42-8 | 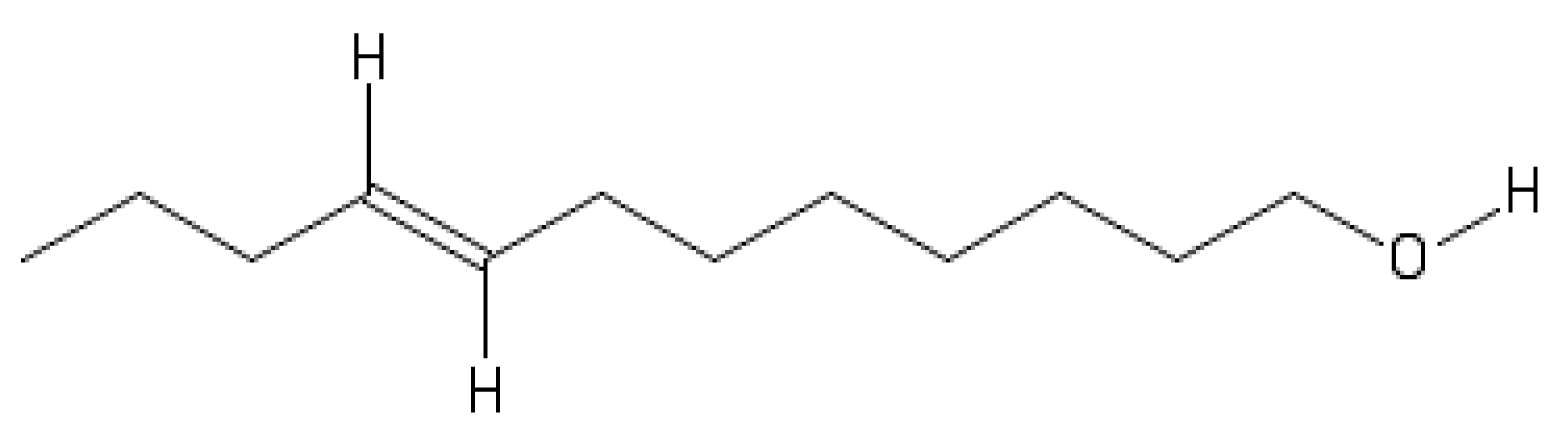 | -6.5 |
| 21 | (E)-linalool oxide | 39028-58-5 | 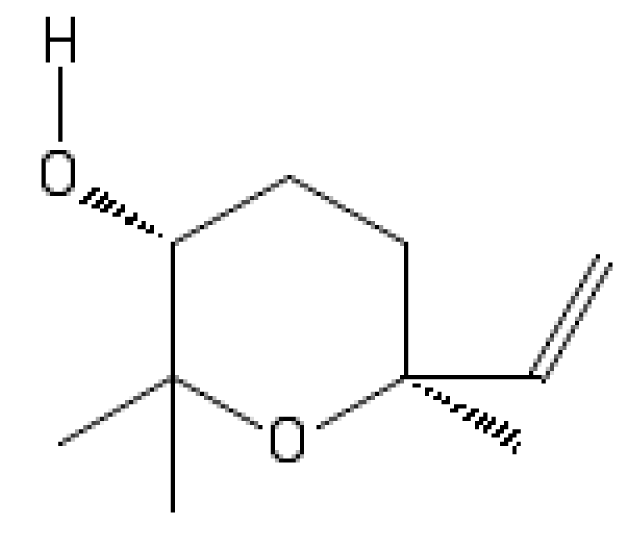 | -6.4 |
| 22 | (E)-Furanoid linalool oxide | 34995-77-2 | 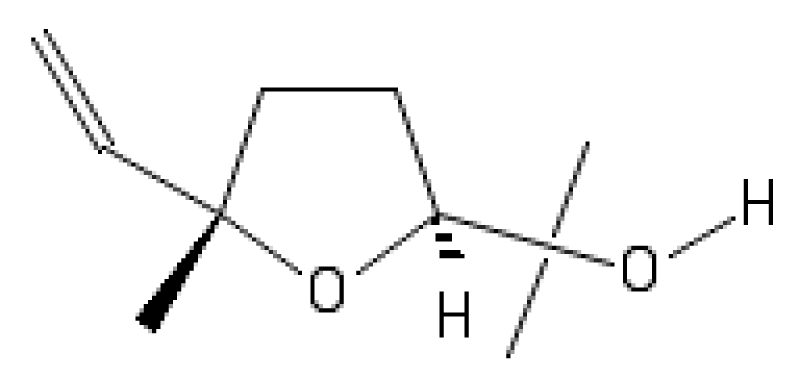 | -6.4 |
| 23 | 1-Dodecanol | 27342-88-7 | 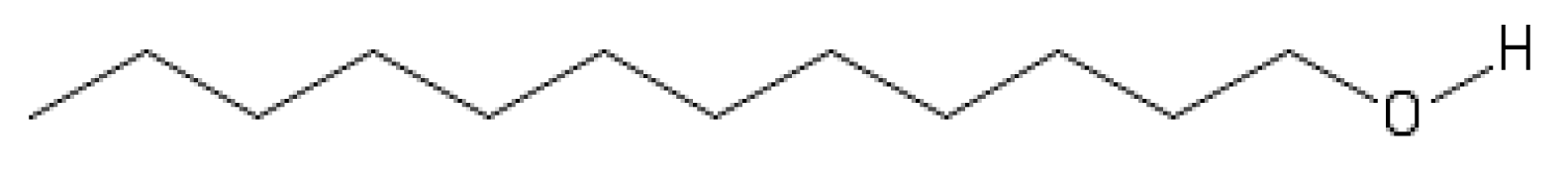 | -6.4 |
| 24 | Z-3-hexenyl-2-methylbutanoate | 53398-85-9 | 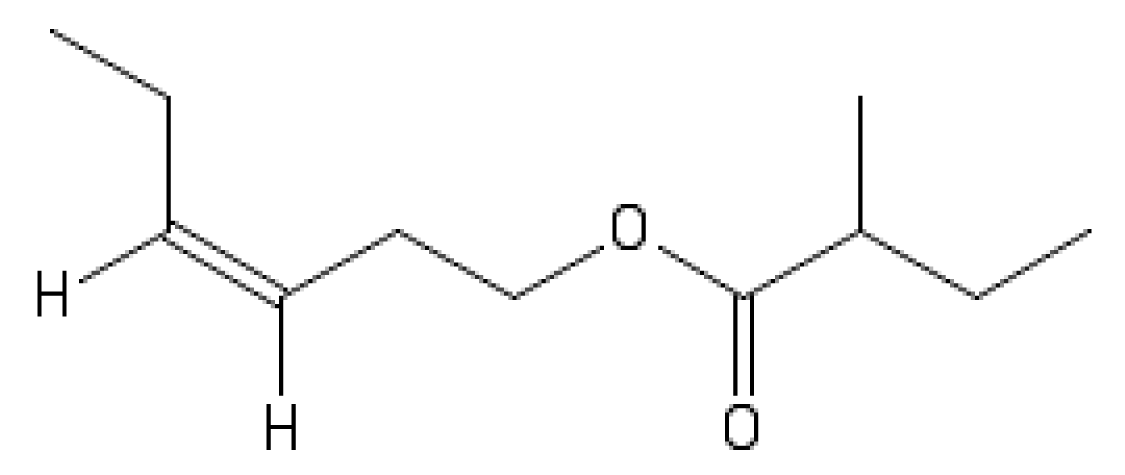 | -6.4 |
| 25 | Isopentyl hexanoate | 2198-61-0 | 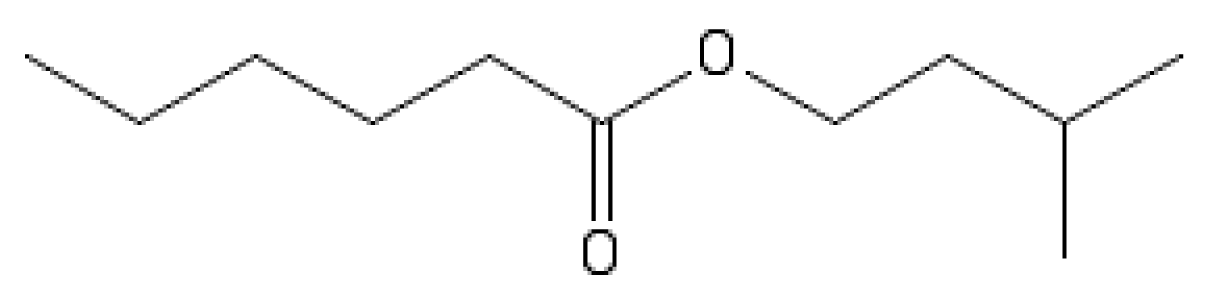 | -6.3 |
| 26 | Methyl salicylate | 119-36-8 | 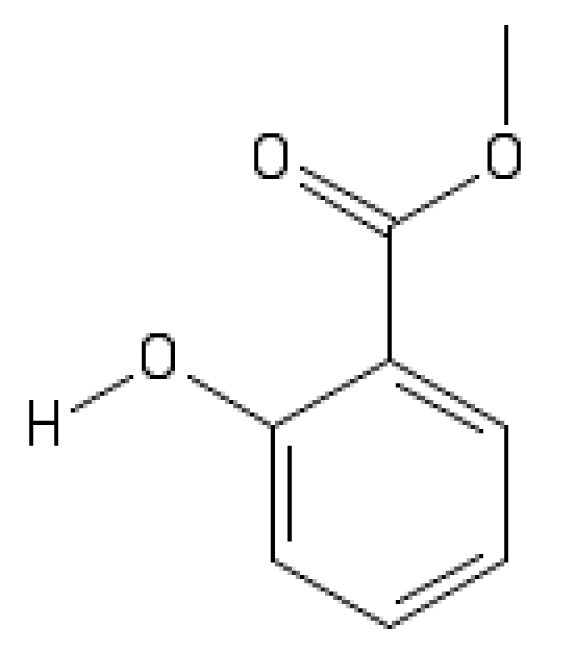 | -6.3 |
| 27 | 3,7-dimethyl-1,6-octadien-3-ol | 78-70-6 | 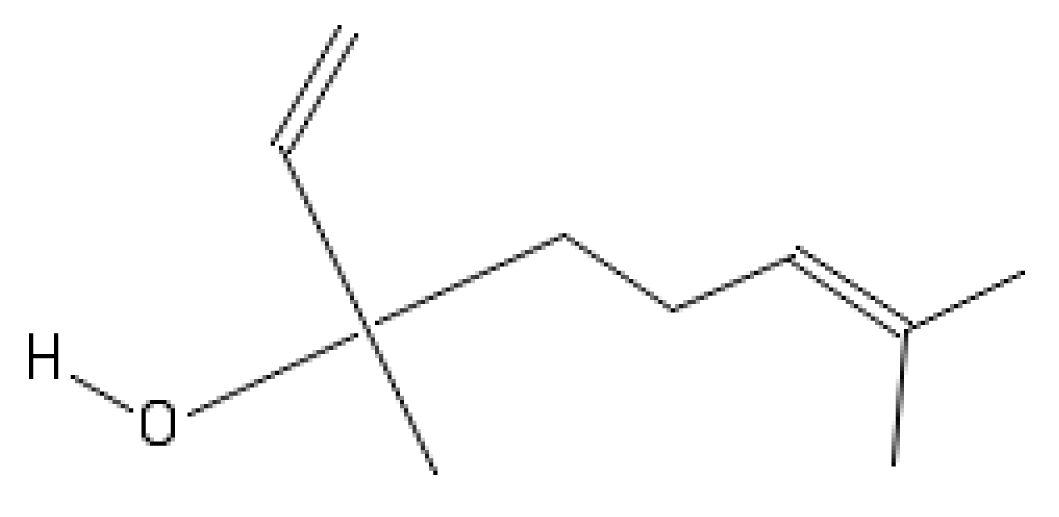 | -6.3 |
| 28 | (Z)3-Hexenyl hexanoate | 31501-11-8 | 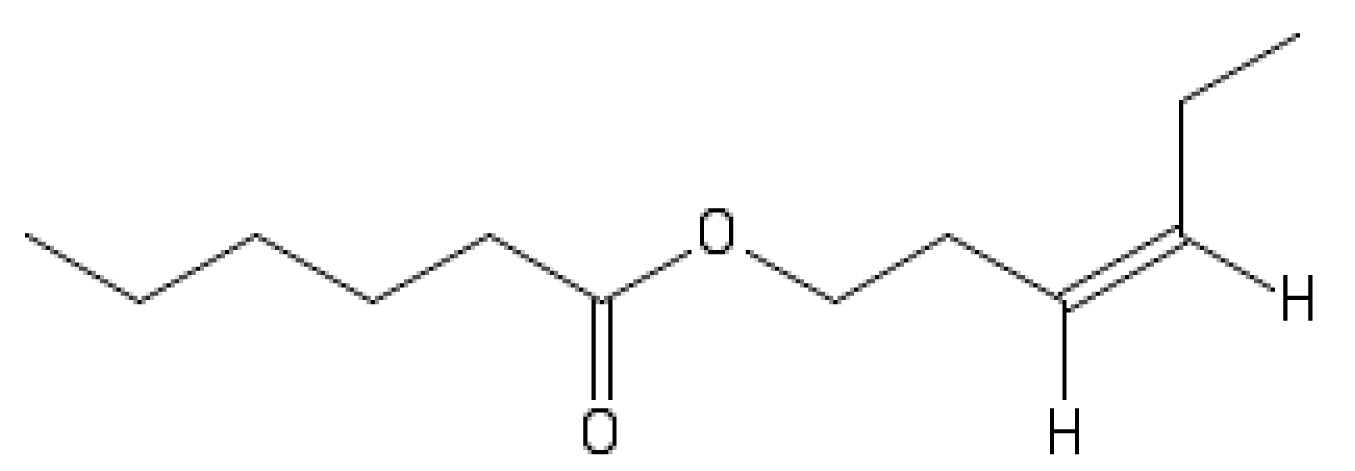 | -6.2 |
| 29 | Hexyl hexanoate | 6378-65-0 | 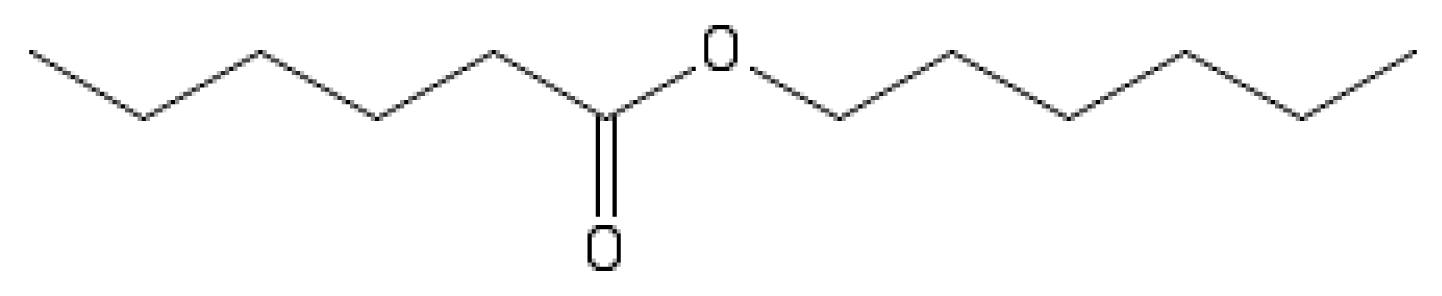 | -6.1 |
| 30 | Hexyl 2-methyl-butanoate | 10032-15-2 | 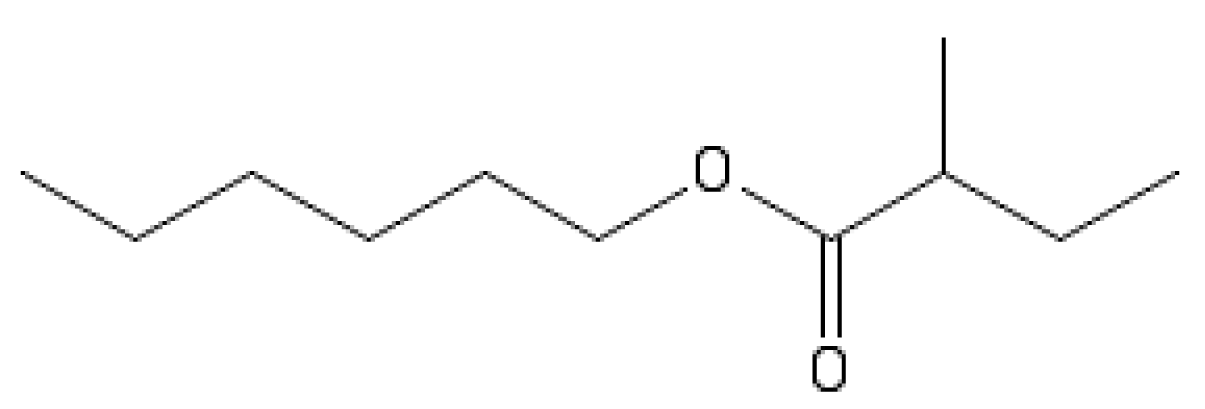 | -6 |
| 31 | Isobutyl hexanoate | 105-79-3 | 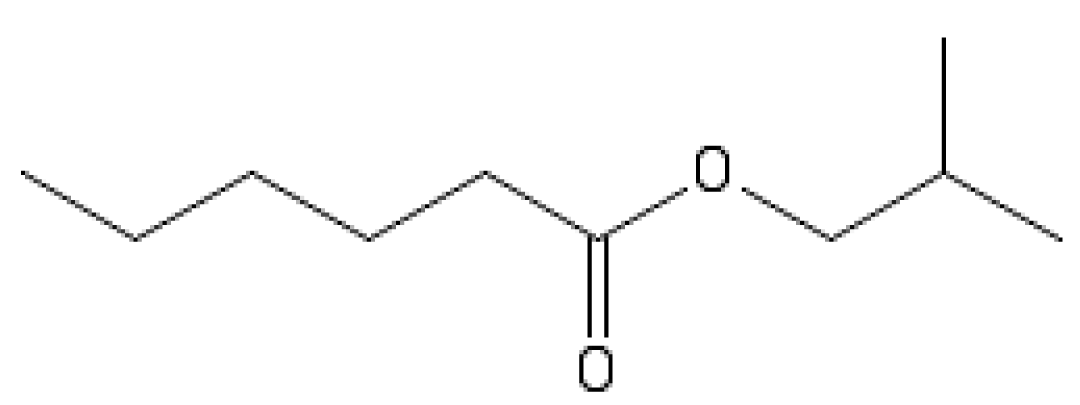 | -6 |
| 32 | Butyl hexanoate | 626-82-4 | 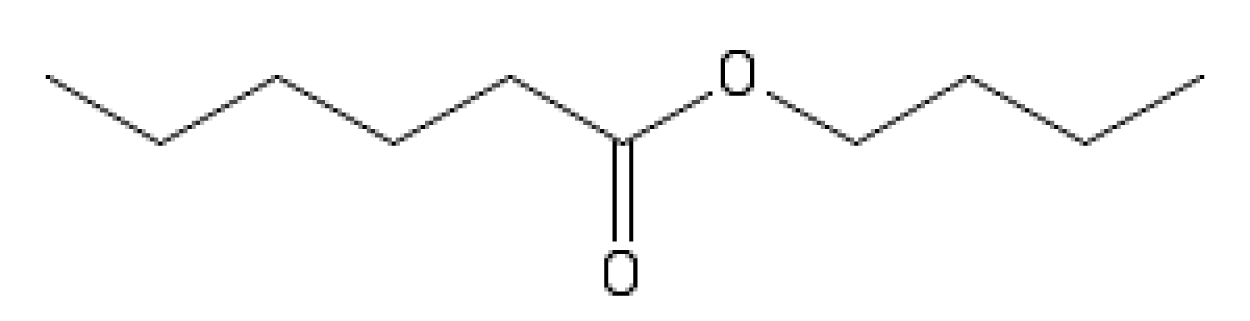 | -5.9 |
| 33 | Hexyl butanoate | 2639-63-6 | 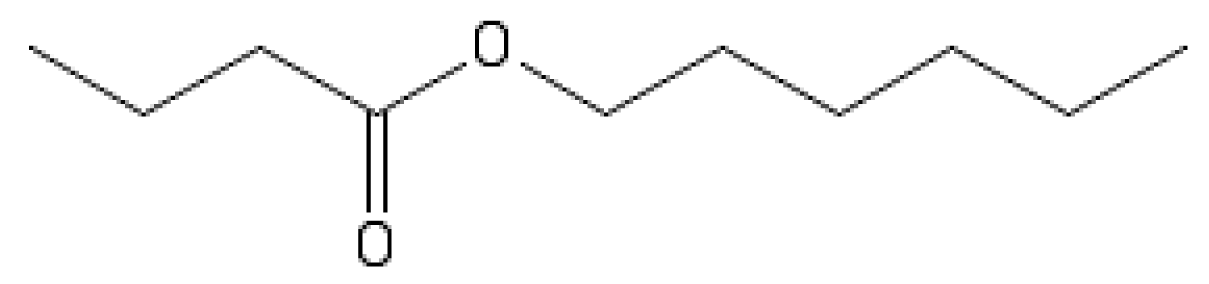 | -5.9 |
| 34 | Benzyl alcohol | 100-51-6 | 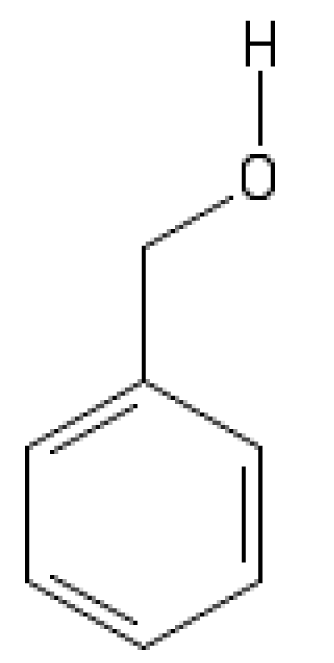 | -5.8 |
| 35 | Hexyl propanoate | 2445-76-3 | 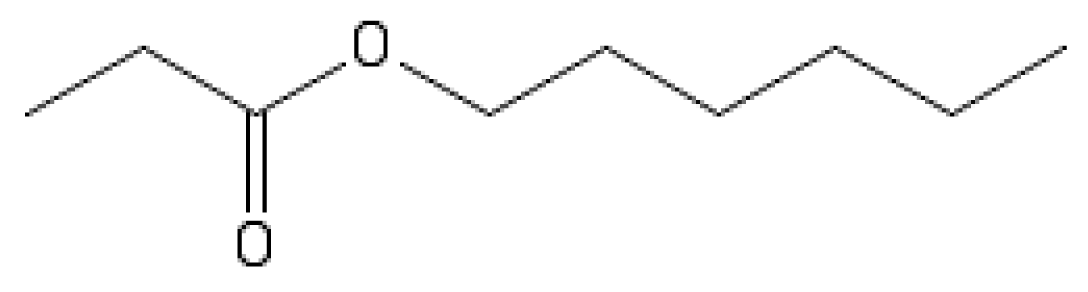 | -5.7 |
| 36 | Z-3-hexenyl acetate | 3681-71-8 | 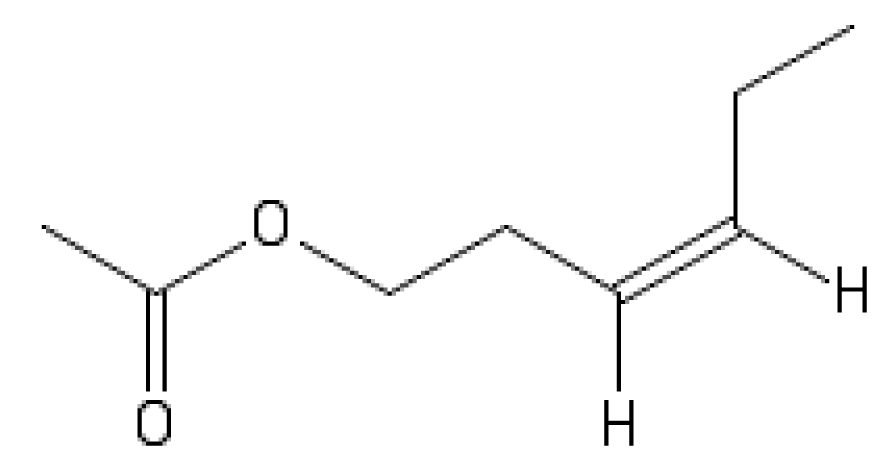 | -5.6 |
| 37 | Propyl hexanoate | 626-77-7 | 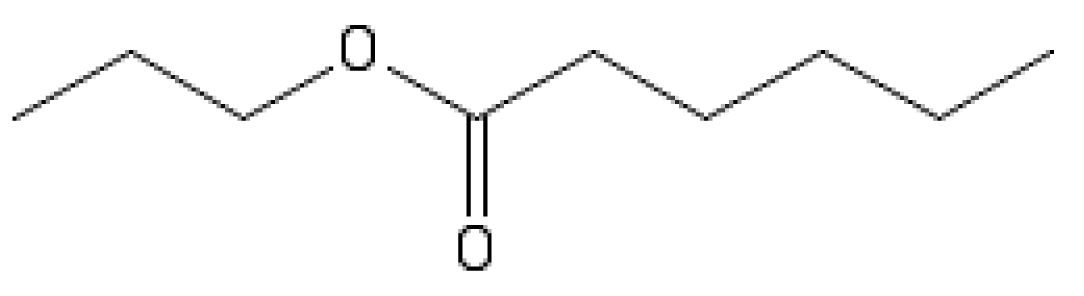 | -5.6 |
| 38 | Hexyl acetate | 142-92-7 | 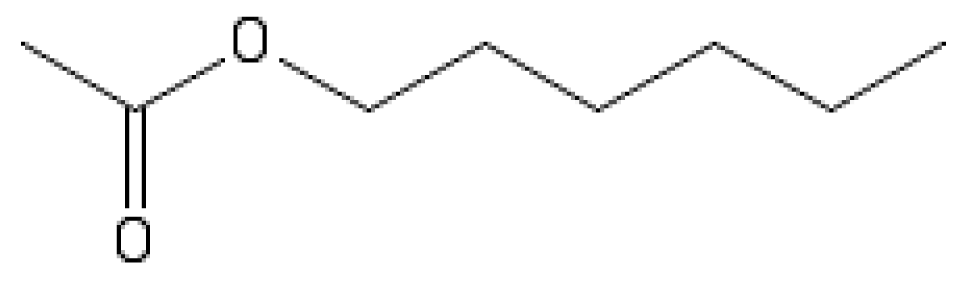 | -5.5 |
| 39 | Pentyl butyrate | 540-18-1 | 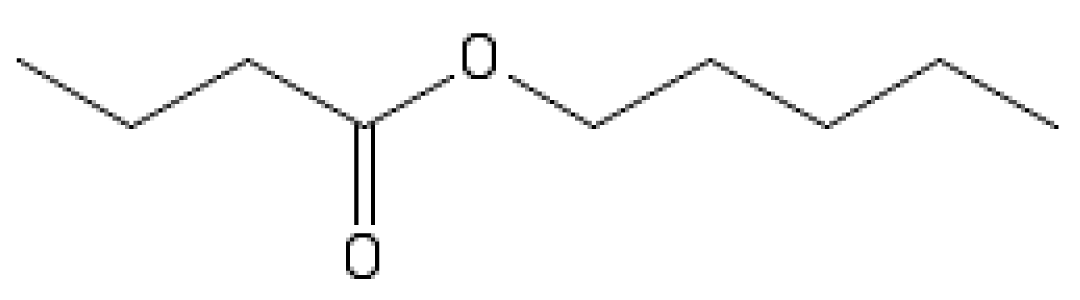 | -5.5 |
| 40 | E-2-hexenyl acetate | 2497-18-9 | 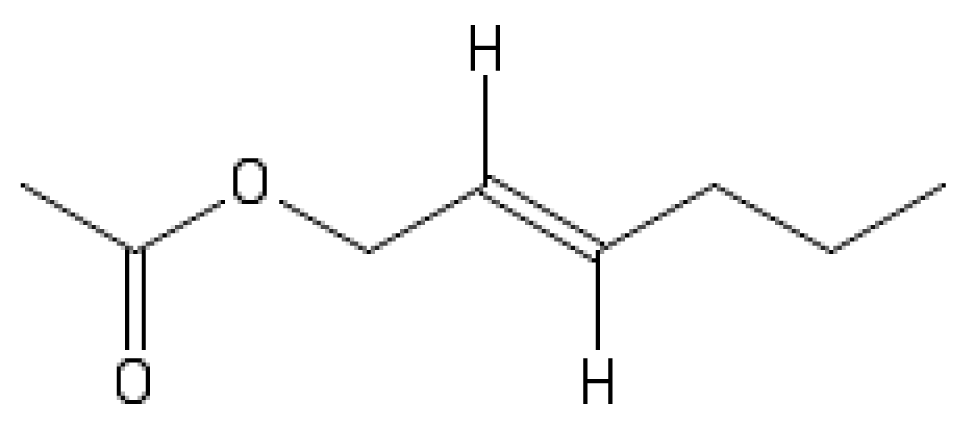 | -5.5 |
| 41 | Butyl valerate | 591-68-4 | 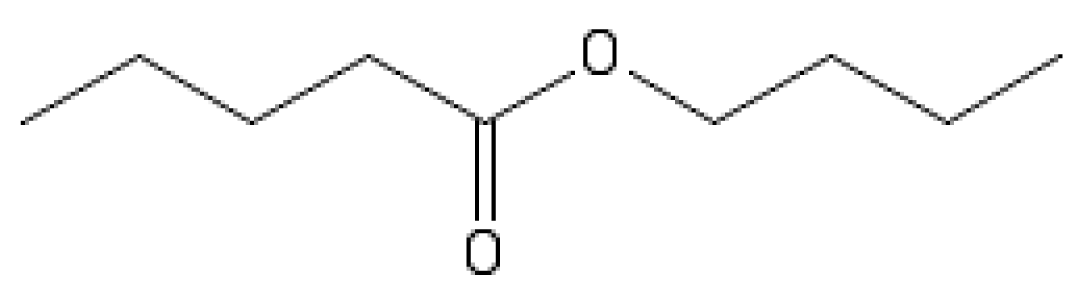 | -5.4 |
| 42 | 2-methyl-butyl acetate | 624-41-9 | 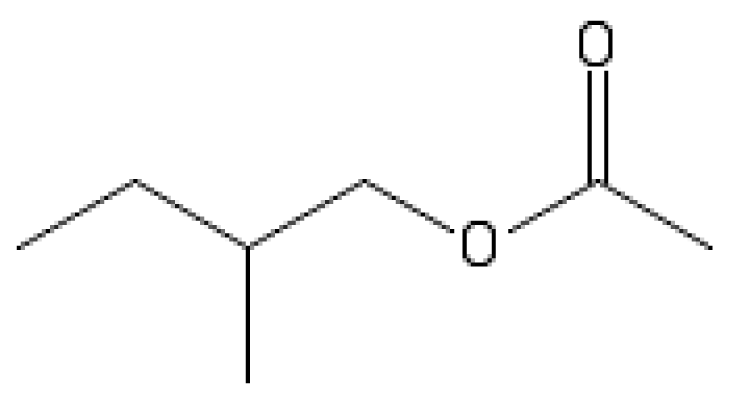 | -5.3 |
| 43 | Butyl butanoate | 109-21-7 | 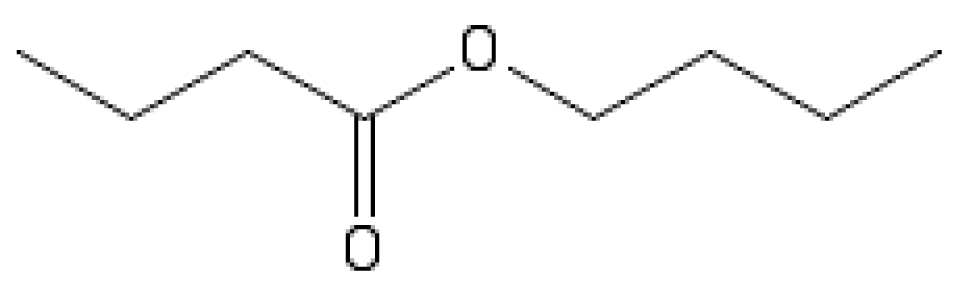 | -5.2 |
| 44 | Butyl butyrate | 109-21-7 | 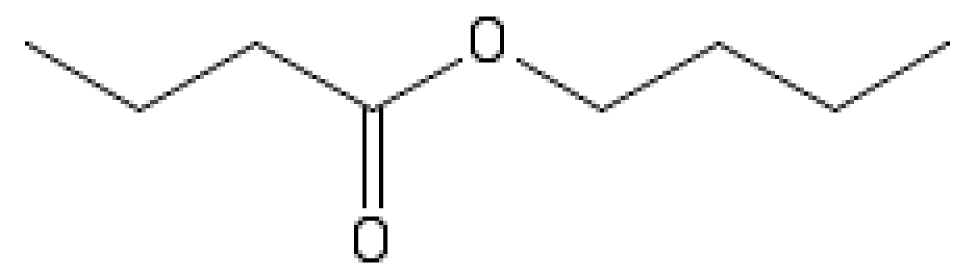 | -5.2 |
| 45 | Ethyl-2-methylbutyrate | 7452-79-1 | 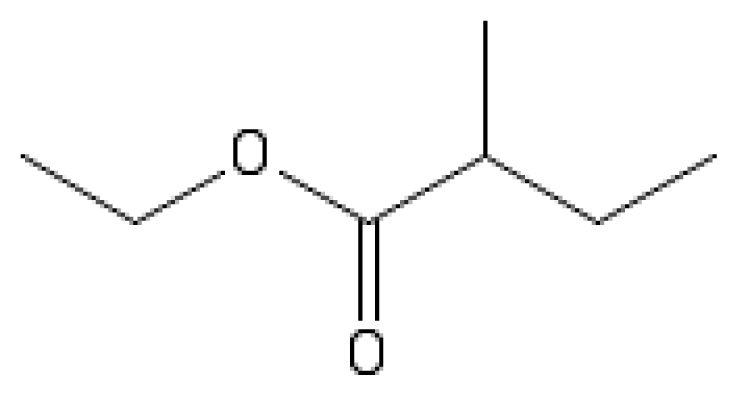 | -5.1 |
| 46 | E-3-hexenol | 928-97-2 | 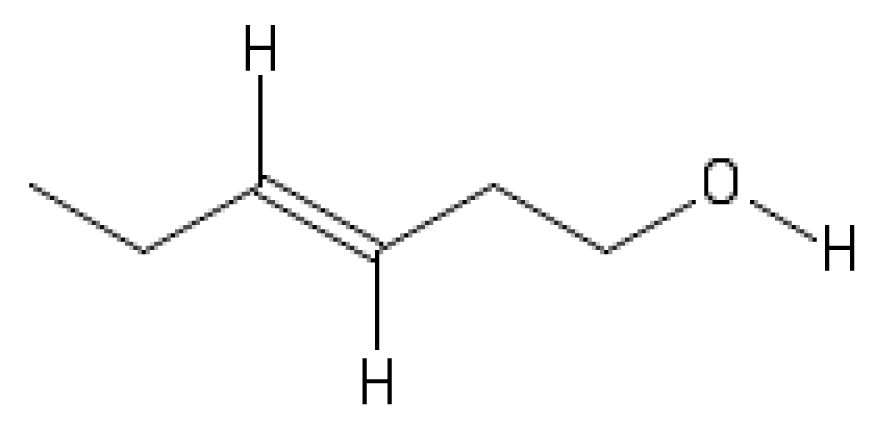 | -4.8 |
| 47 | (Z)3-hexenol | 928-96-1 | 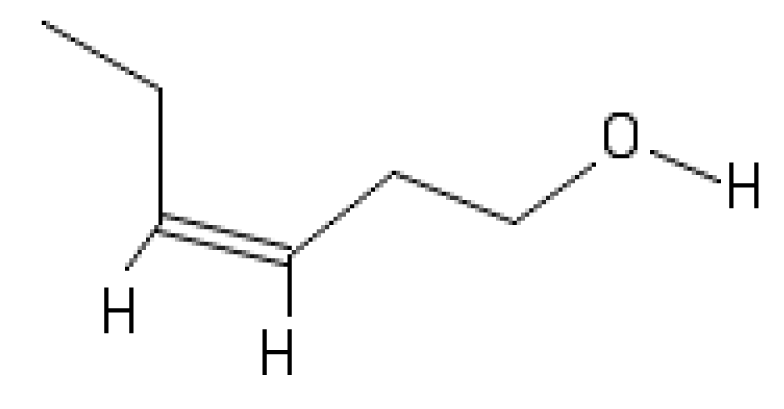 | -4.8 |
| 48 | acetic acid (AA) | 64-19-7 | 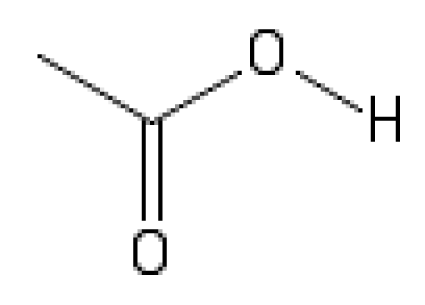 | -2.9 |
